# The relative influence of sea surface temperature anomalies on the benthic composition of an Indo‐Pacific and Caribbean coral reef over the last decade

**DOI:** 10.1002/ece3.9263

**Published:** 2022-09-06

**Authors:** Jack V. Johnson, Dan A. Exton, Jaimie T. A. Dick, Joseph Oakley, Jamaluddin Jompa, Daniel Pincheira‐Donoso

**Affiliations:** ^1^ Macrobiodiversity Lab, School of Biological Sciences Queen's University Belfast Belfast UK; ^2^ Operation Wallacea Spilsby UK; ^3^ Institute for Global Food Security, School of Biological Sciences Queen's University Belfast Belfast UK; ^4^ Independent Researcher London UK; ^5^ Graduate School Hasanuddin University Makassar Indonesia

**Keywords:** Anthropocene, global warming, Honduras, reef composition, refuge, resistance, Wakatobi

## Abstract

Rising ocean temperatures are the primary driver of coral reef declines throughout the tropics. Such declines include reductions in coral cover that facilitate the monopolization of the benthos by other taxa such as macroalgae, resulting in reduced habitat complexity and biodiversity. Long‐term monitoring projects present rare opportunities to assess how sea surface temperature anomalies (SSTAs) influence changes in the benthic composition of coral reefs across distinct locations. Here, using extensively monitored coral reef sites from Honduras (in the Caribbean Sea), and from the Wakatobi National Park located in the center of the coral triangle of Indonesia, we assess the impact of global warming on coral reef benthic compositions over the period 2012–2019. Bayesian generalized linear mixed effect models revealed increases in the sponge, and hard coral coverage through time, while rubble coverage decreased at the Indonesia location. Conversely, the effect of SSTAs did not predict any changes in benthic coverage. At the Honduras location, algae and soft coral coverage increased through time, while hard coral and rock coverage were decreasing. The effects of SSTA at the Honduras location included increased rock coverage, but reduced sponge coverage, indicating disparate responses between both systems under SSTAs. However, redundancy analyses showed intralocation site variability explained the majority of variance in benthic composition over the course of the study period. Our findings show that SSTAs have differentially influenced the benthic composition between the Honduras and the Indonesian coral reefs surveyed in this study. However, the large intralocation variance that explains the benthic composition at both locations indicates that localized processes have a predominant role in explaining benthic composition over the last decade. The sustained monitoring effort is critical for understanding how these reefs will change in their composition as global temperatures continue to rise through the Anthropocene.

## Graphical abstract

Global warming continues to transform coral reef assemblages throughout the tropics, impacting biodiversity and ecosystem function. Here we identify disparate disruptions between an Indoneisan coral reef and a Honduran coral reef system under climate change over the last 10 years. " cd_value_code="text

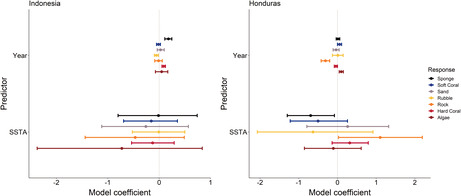

## 1 INTRODUCTION

Coral reefs harbor the highest levels of biodiversity of all marine ecosystems (Fisher et al., 2015), performing paramount roles in the stability of ocean life (Benkwitt et al., 2020; Oliver et al., 2015). In addition, the extraordinary complexity of coral reefs sustains a range of key ecosystem services to human wellbeing, including food security, storm protection, and economic benefits relevant to hundreds of millions of people around the globe (Foale et al., 2013; Moberg & Folke, 1999; Norström et al., 2016; Woodhead et al., 2019). However, rising ocean temperatures linked to increased anthropogenic emissions of greenhouse gasses have been identified as a key threat to coral reef persistence (Hughes et al., 2017).

A robust body of evidence has shown that global warming acts as the key driver of coral reef declines throughout the tropics. Pulse events such as marine heatwaves are widely documented to induce bleaching of corals, a process where photosynthetic endosymbionts are expelled from the cnidarian host (Boilard et al., 2020; Douglas, 2003; Fitt et al., 2001; Suggett & Smith, 2020; Warner et al., 1999). Bleaching is occurring over large spatial scales, resulting in mass mortality of entire coral colonies (Hughes, Anderson, et al., 2018; Hughes, Kerry, et al., 2018). Additionally, the continued rise in ocean temperatures is preventing coral reefs from recovering before further pulse events occur (Harrison et al., 2019; Hughes, Anderson, et al., 2018). Rising ocean temperatures also inhibit the recruitment of coral reefs by causing mortality to juvenile corals (Hughes et al., 2019), highlighting the multifaceted process of coral reef decline via global warming. Thus, global warming will continue to transform coral reefs into taxonomically, physically, and functionally more homogenous environments (Hughes, Kerry, et al., 2018), reducing biodiversity and impacting ecosystem function (Brandl et al., 2019; Oliver et al., 2015; Pratchett et al., 2011).

As global warming continues to degrade coral reefs across the globe, monopolization by other taxa such as macroalgae where reef corals previously resided can occur rapidly (Bozec et al., 2019; Fulton et al., 2019; Graham et al., 2013; Hughes et al., 2007). Additionally, other taxa may also monopolize space previously inhabited by hard corals, such as sponges (Bell et al., 2013; Lesser & Slattery, 2020; Pawlik et al., 2016) and soft corals (Inoue et al., 2013). Yet these taxa do not provide equal ecological complexity to support biodiversity and provision of ecosystem services as reef‐building corals (Friedlander & Parrish, 1998; Hughes et al., 2017; Woodhead et al., 2019). Furthermore, a combination of biotic interactions and abiotic effects can prevent taxa from monopolizing uninhabited space for a period of time, resulting in an increased prevalence of sand or rock across the reef scape, further reducing habitat heterogeneity (Alvarez‐Filip et al., 2009). Finally, other nonliving benthic components such as coral rubble can inhabit reef space, a clear indication of hard coral mortality, and thus substratum homogenization. These changes in benthic and taxonomic compositions of coral reefs ultimately represent a phase shifts of coral reefs, which are becoming more common under global warming in the Pacific (Bozec et al., 2019; Ledlie et al., 2007), along the Great Barrier Reef (Hughes et al., 2007) and especially in the Atlantic Ocean (Roff & Mumby, 2012).

Coral reefs in the Wakatobi National Park (WNP) of Indonesia, and Honduras in the Caribbean, represent two extensively monitored locations since 2012, providing an ideal case study for understanding long‐term benthic compositional change under sea surface temperature anomalies (SSTAs). In the Honduran reef systems, coral cover has been stable between sites (Titus et al., 2015). Meanwhile, depths between 5 and 15 m are associated with divergent responses between hard coral and macroalgae cover, but not sponge and soft coral cover at Utila, an island north of the Honduras coast (Andradi‐Brown et al., 2016). At the Indonesia location, fine‐scale site variability has been reported for key benthic components, such as Sponge dominance on the turbid reefs (Biggerstaff et al., 2017; Powell et al., 2014; Rovellini et al., 2019), while algae coverage shows temporal variability across reefs at this location (Marlow et al., 2020). By contrast, hard coral cover has appeared relatively stable at the WNP (Marlow et al., 2020), despite observed general global declines since the turn of the century owing to anthropogenic heating (Bruno & Selig, 2007). However, coral community composition did change in the WNP, with a reduction of ~20% in hard coral cover linked to an intense bleaching event in 2010 (Watt‐Pringle et al., 2022).

While previous findings have identified spatial and temporal variations of benthic cover at these extensively monitored locations, the change in benthic composition has not been assessed with satellite‐derived temperature metrics related to SSTAs, Here we assess the relative role of elevated sea temperatures from remote sensing data for influencing the benthic composition two coral reefs from distinct bioregions from 2012 to 2019.

## 2 METHODS

### 2.1 Survey locations

**FIGURE 1:**
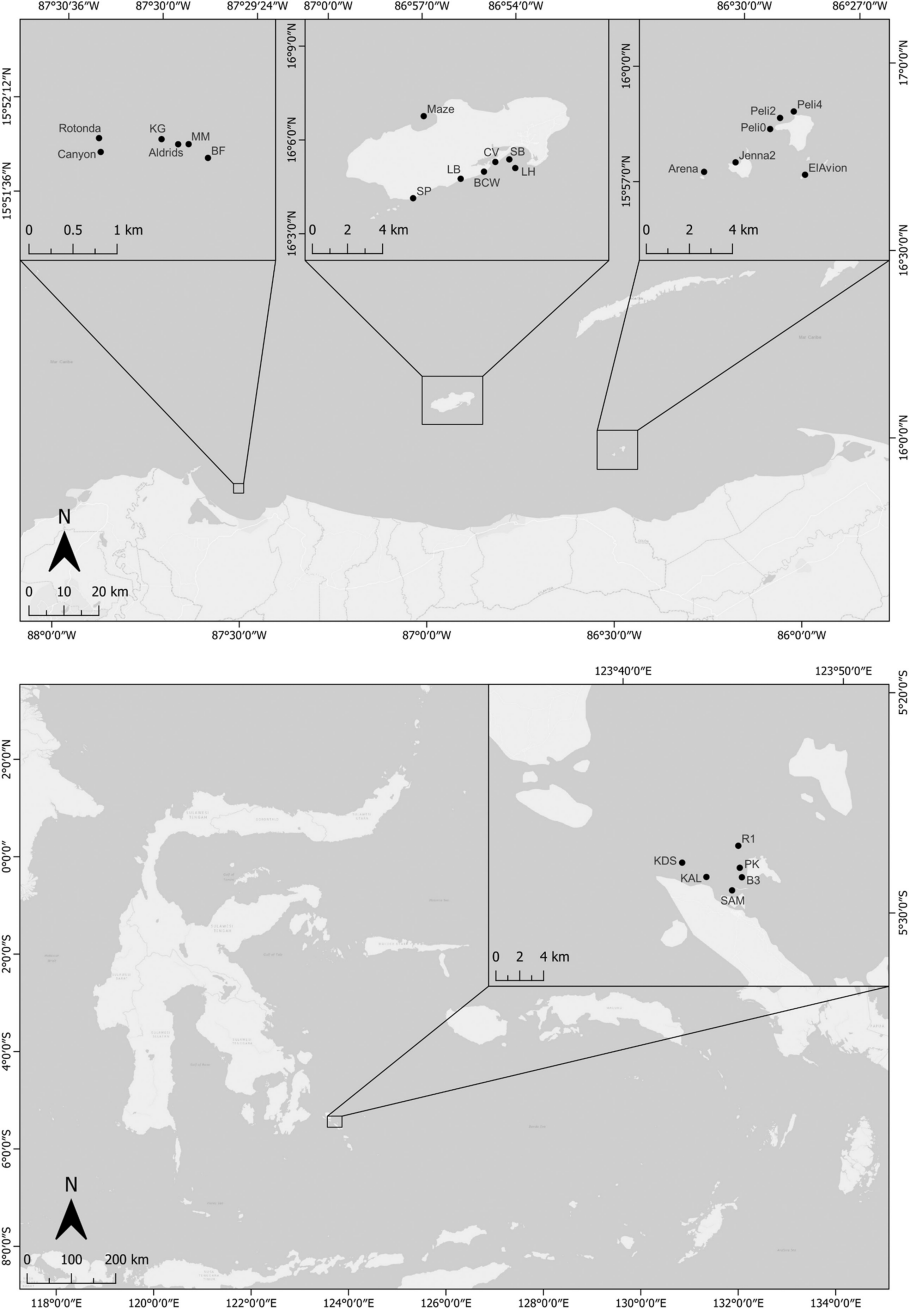
Locations of reefs where field surveys were undertaken for benthic data collection by Operation Wallacea citizen scientists. The top panel shows the location of surveyed reef sites from Honduras; the bottom pannel shows the surveyed reef sites of Indonesia, located in the WNP.

Our study aims to compare two major coral reef systems of Honduras and Indonesia (Figure 1) where long‐term monitoring by Operation Wallacea has been carried out.

In Honduras, data were collected from multiple reef sites in three distinct locations. Cayos Cochinos Marine Protected Area is a small archipelago close to the Honduran mainland with an extensive network of gently sloping coral reefs and heavily restricted access (Titus et al., 2015). Utila Island is the smallest of the Bay Islands chain and home to a major dive tourism industry and surrounded by a fringing reef ranging from slopes to steeper walls (Andradi‐Brown et al., 2016). Finally, Banco Capiro is a recently discovered reef system in the mainland bay of Tela, comprising an offshore bank that is home to an unusually high percentage cover of live coral for the region (Bodmer et al., 2015) and a uniquely high‐density population of the keystone herbivorous urchin *Diadema antillarum* (Bodmer et al., 2021).

The study sites in Indonesia were located in the WNP, South‐east Sulawesi. The park encompasses 1.39 million hectares (https://wakatobinationalpark.id/peta‐kerja/) in the center of the Coral Triangle, harboring over 390 species of hard coral, and 590 fish species across the 50 k hectares of coral reefs (Clifton et al., 2010). Approximately 100 k people reside within the WNP, many of which directly rely on coral reefs for their daily livelihoods (Cullen et al., 2007; Exton et al., 2019). Monitoring efforts in the WNP have focused on reefs from Kaledupa Island, and the smaller adjacent Hoga Island (Figure 1). Surveys were taken across six established study sites encompassing various types of coral reefs. Buoy 3 and Ridge 1 are steep‐walled sites, Pak Kasims, Kaledupa, and KDS are gentle slope reefs, while Sampela is a gentle sloped highly sedimented and turbid reef (Crabbe & Smith, 2002; Marlow et al., 2018).

### 2.2 Benthic data

Benthic surveys took place during the months of June, July, and August from 2012 to 2019. The six reef sites in Indonesia were replicated each year, while multiple sites in Honduras were randomly surveyed throughout the distinct locations over the 8‐year study period. Benthic data were collected by trained underwater surveyors using SCUBA. Survey teams were made up of university‐level volunteers led by trained experienced scientists in underwater surveying. The standardized methodology required surveyors to perform 50 m line intercept transects, where data were collected every 0.25 m along the transect, recording data on benthic biotic or abiotic classification under the transect tape at that point. Transects were replicated at 5, 10, and 15 m at the Honduras location only. Whereas at the Indonesia location, surveys were triplicated at the key reef zones, being the reef crest (~5 m), the reef flat (~2–3 m), and the reef slope (~12–15 m). Using a variety of depths at the Honduras sites, a variety of reef zones at the Indonesia sites, which encompasses a wide depth range of shallow reefs (2–15 m), allows us to generalize the shallow reef benthic compositions at these sites. Categories for the benthic classifications are identified in Table 1.

**TABLE 1:** Categorization of biotic and abiotic benthic components collected from benthic transect surveys

Code	Benthic category
AL	Algae
AN	Anemone
ASC	Ascidian
CCA	Coralline Crustose Algae
DC	Dead Coral
HC	Hard Coral
HYD	Hydroids
INV	Other Invertebrate
MI	Millipora
PEY	Peysonnellia
RB	Rubble
RCK	Rock
S	Sand
SC	Soft Coral
SG	Sea Grass
SI	Silt
SP	Sponge
UN	Unknown
W	Water
ZO	Zooanthid

### 2.3 Environmental data

Heat stress was quantified as SSTAs measured in °C over the last 52 weeks preceding surveying at a 5‐km resolution, extracted from Coral Reef Watch (CRW) v3.1 5‐km product suite (Liu et al., 2014). The 5‐km daily SSTA product uses the daily climatology (DC) derived from the monthly mean (MM) climatology interpreted from linear interpolation. The MM value is assigned to the 15th day of each corresponding month, where individual days are derived from the linear interpolation. The SSTA value is thus calculated as follows

SSTA=SST−DC

where the SST (sea surface temperature) is the value for the day, and DC is the corresponding DC for that specific day of the year.

The CRW products are highly robust for accurately measuring thermal stress, especially in tropical latitudes (Liu et al., 2014), with many different products utilized for various types of study (e.g., Hughes, Kerry, et al., 2018; McClanahan et al., 2019, 2020). Given the discrepancies in accuracy between satellite‐derived temperature data and actual temperature of a given region, small values between −0.2 and +0.2°C are considered climatologically normal for the SSTA product, exemplifying the robustness of CRW data (Liu et al., 2014). These satellite‐derived temperature data are primarily an excellent tool for predicting coral responses to heat stress in shallow water (Johnson et al., 2022a, 2022b; Sully et al., 2019). Additionally, they are also ideal predictors of coral responses of up to 18 m depth for changes in coral assemblages (Hughes, Kerry, et al., 2018), and coral mortality (Donovan et al., 2021).

**FIGURE 2:**
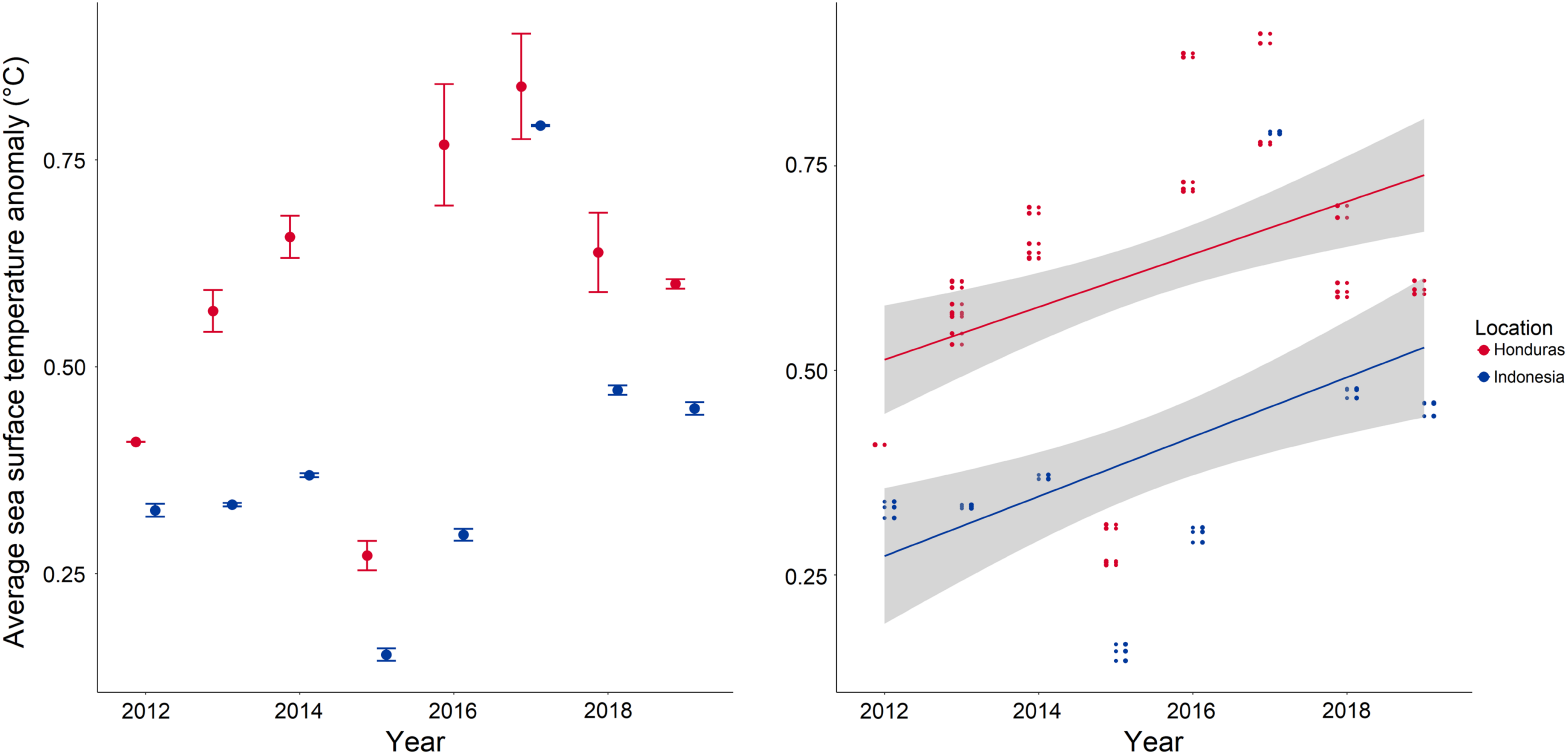
Average sea surface temperature anomaly (SSTA) in °C and standard errors (whiskers) from 2012 to 2019 at the Indonesia and Honduras sites surveyed in this study. Temperatures were quantified from the 52 weeks preceding the survey period, which began at the June 1 for each site, each year. Points represent the mean SSTA, while error bars are standard error.

The CRW product SSTA values were extracted as summarized values on a weekly time series. SSTA values were extracted at a 5‐km resolution for each location over the previous 52 weeks from when the benthic surveying commenced (i.e., June 1st–May 31st) for each year from 2012 to 2019. Benthic surveys where SSTA data were not available were excluded from the analysis, leaving a total of 1088 surveys over the 8‐year time period. While there are potential issues (Ferguson et al., 2017) for using mismatched time series (i.e., values over the last 52 weeks before the commencement of the survey), which do not capture fine‐scale variability taxa with fast life histories, such as macroalgae and some sponges (Rovellini et al., 2021), this approach has been successfully employed for assessing coral responses over the time period used (Donovan et al., 2021), which are indicative of coral reef compositional change. Average SSTA values for each location over the course of the previous 52 weeks are summarized in Figure 2. The number of temperature cells used to extract temperature values for the surveys from CRW at each location is in Table 2.

**TABLE 2:** The Coral Reef Watch (CRW) temperature cells used for each site at each location in the study

Location	Sties sharing a CRW cell
Indonesia	B3 and PK
	Sampela, KDS, and KAL
	R1
Honduras	BCW, CV, LB, and SB
	LH
	El Avion, Peli0, Peli2, and Peli4
	SP
	Maze
	BF, KG, MM, Aldrids
	Canyon, Rotanda
	Arena
	Jenna 2

### 2.4 Statistical analyses

Firstly, a generalized linear model (GLM) with a quasi‐Poisson distribution was used to determine whether the average SSTA was increasing through time at each location, as data were Poisson‐distributed and over‐dispersed.

#### 2.4.1 Bayesian generalized linear models

To assess the response of benthic components to rising ocean temperatures, we used Bayesian GLMs from the “brms” package (Bürkner, 2017), which utilizes the STAN language (Carpenter et al., 2017) in R 4.1.0 (R Core Team, 2021). The cover of each benthic component was run as a response variable at each location (Indonesia and Honduras), specified with beta distribution, as survey data reflected proportions. Time (the year of survey—2012) and SSTA were the explanatory effects in the model, run with the random effect of site. Priors were fitted for each model using the “*get_prior*” function in the package “brms,” which specifies priors for the beta coefficients, intercept, and random of effects of each model (Bürkner, 2017). Models were run for 4000 iterations with 3000 burnins, across four chains. To ensure convergence was achieved trace plots were assessed (Figures S1‐S14). Posterior predictive checks were also used to assess model performance (Figures S1‐S14), in addition to each model achieving a Gelman‐Rubin statistic (Rhat) of 1 (Bürkner, 2017).

#### 2.4.2 Ordination analysis

To assess the relative influence of our predictors of time and SSTA for predicting benthic composition at each location over the entire survey period (2012–2019) we used Redundancy analysis (RDA) from the “vegan” package (Oksanen et al., 2013) in R. RDA is analogous of ordinary least square regression to the multivariate response variable, which expects a linear response of each benthic component to the environmental variables (Year, SSTA, and site). Using RDA allowed us to extract the constrained inertia (variance explained) from each model at the two locations to assess the relative influence of time and SSTA on driving benthic composition.

Further analysis to assess changes in the benthic components of coral reefs our locations were assessed using non‐Metric Multi‐Dimensional Scaling (nMDS) from the “vegan” package (Oksanen et al., 2013). Each benthic component was ordinated in 2‐dimensional space grouped by the first 4 years of sampling (2012–2015) and the last 4 years of sampling (2016–2019) with a Euclidean dissimilarity matrix. Grouping the composition of coral reefs this way coincided with the 2016–2017 back‐to‐back bleaching events where marine temperatures were exceedingly high (Hughes, Anderson, et al., 2018), devastating many corals around the globe (Harrison et al., 2019; Hughes, Anderson, et al., 2018; McClanahan et al., 2019; Sully et al., 2019), even leading to transformed coral reef assemblages on the Great Barrier Reef (Hughes, Kerry, et al., 2018). To assess the change in the composition of each site between the two grouped time periods, the entirety of the ordination space occupied by each location was plotted, along with their pairwise distances.

## 3 RESULTS

### 3.1 Sea surface temperature anomalies from 2012 to 2019

The average SSTAs over the last decade were highly divergent between the Honduras and Indonesia locations, with peak SSTAs preceding the survey years of 2016 and 2017 for Honduras (Figure 2). Comparatively, the SSTA peak for Indonesia over the last decade only occurred for 1 year, preceding 2017 surveying (Figure 2). Overall, the average SSTA was higher for every survey year at the Honduras location compared with the Indonesia location. The SSTA also showed a significant increase through time for both the Indonesia location (GLM, Estimate = 3.644, *t* = 0.025, *p* < .001) and the Honduras location (GLM, Estimate = 0.052, *t* = 3.905, *p* < .001).

### 3.2 Response of benthic components to SSTA over the last decade

**FIGURE 3:**
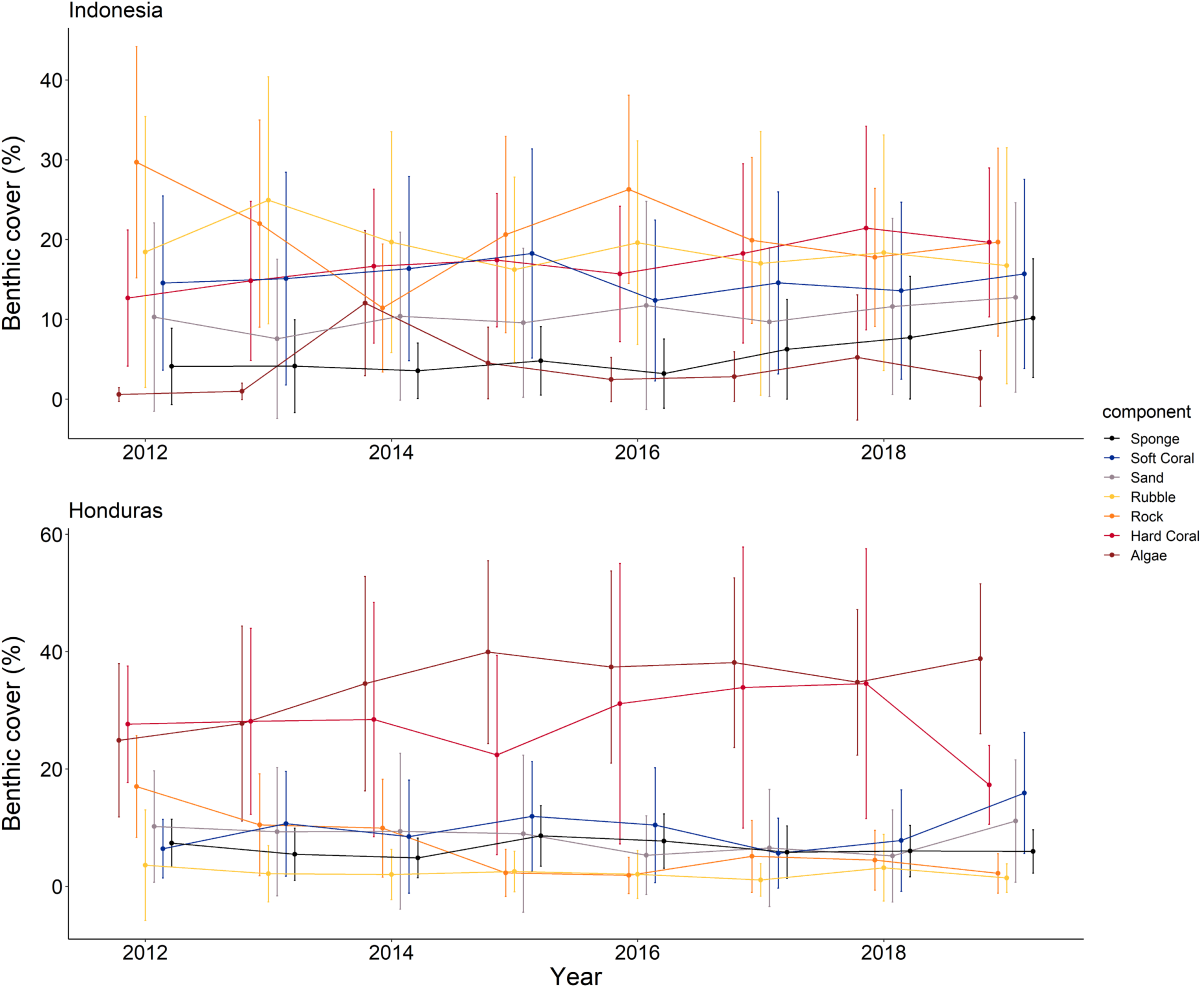
Temporal dynamics of each benthic cover for each category at the Indonesia (top) and Honduras (bottom) location.

**FIGURE 4:**
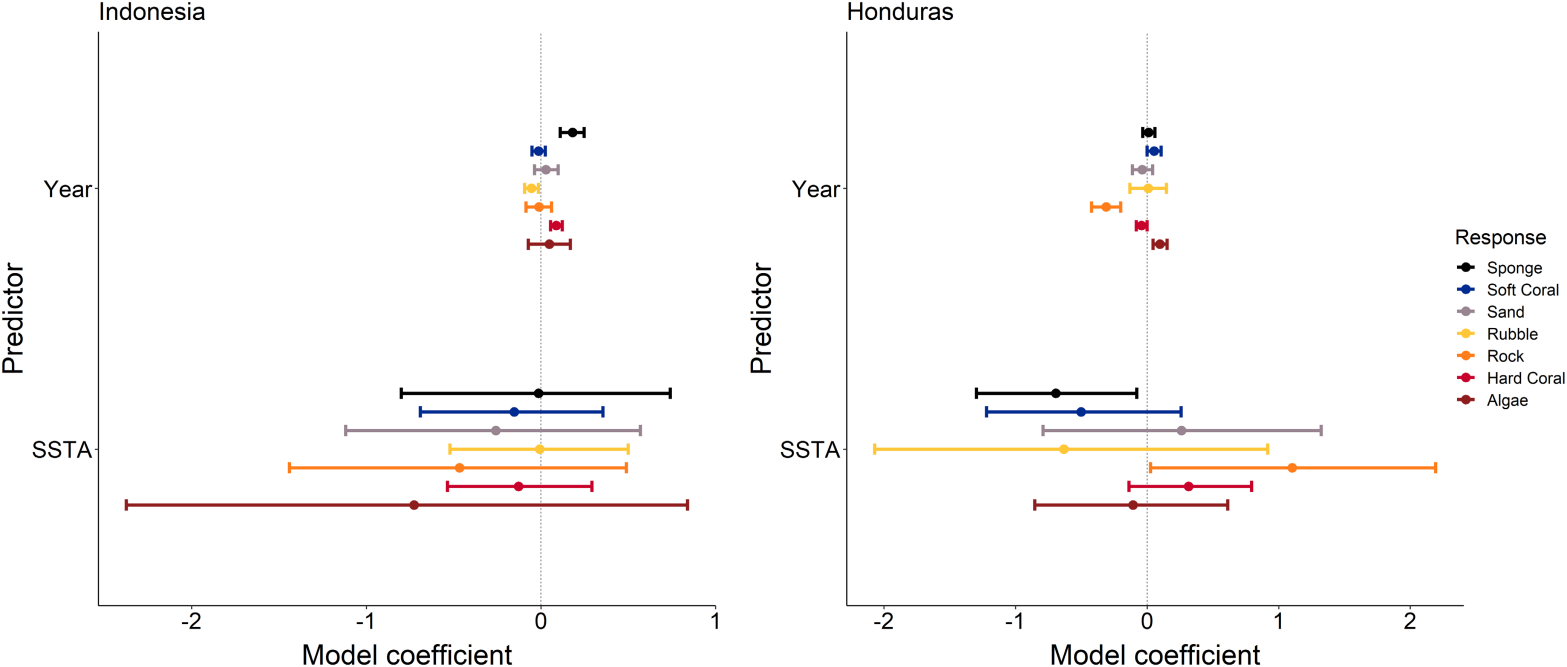
Bayesian GLM coefficient estimates for the response of selected major benthic components under elevated sea surface temperatures (SSTA) and time (Year). Colored points correspond to the specified benthic component, representing the mean model coefficient. Horizontal bars represent 95% credible intervals, which are considered “significant” when they do not cross zero (gray line). The models ran separately for Indonesia and Honduras locations. The components were selected based on preliminary analysis as the most dominant components of reefs from the field surveys undertaken by Operation Wallacea volunteers from the years of 2012 to 2019. Colors are from *Centropyge loricula* using the “fishualize” package (Schiettekatte et al., 2022).

Changes in the benthic composition between locations varied from 2012 to 2019 (Figure 3). Thus, as expected, the response of benthic components to SSTAs also varied between locations (Figure 4). Time (year) was a strong predictor of an increase in hard coral cover and sponge cover at the Indonesia location, while also predicting a decrease in coral rubble. At the Honduras location, the time predicted an increase in algae and soft coral cover. However, hard coral cover and rock cover are predicted to decrease through time. Over the last decade, SSTA did not predict any changes in benthic cover at the Indonesia location. Conversely, SSTA predicted an increase in bare rock cover at the Honduras location, while also predicting a reduction in sponge coverage.

**FIGURE 5:**
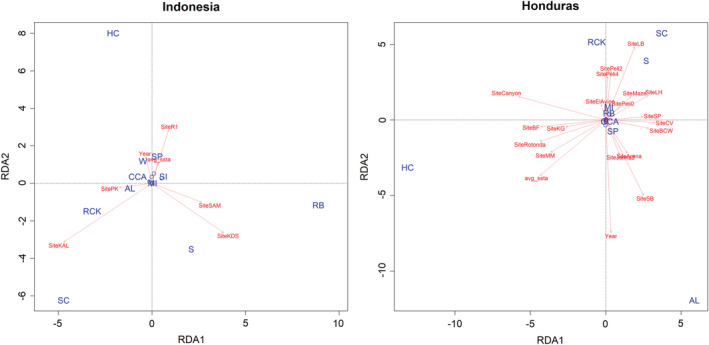
Redundancy analysis of the benthic community composition at each location and their relationship with environmental variables. The left plots are data from Indonesia, while Honduras is shown on the right. The blue text within the plots indicates the individual benthic components (Table 1), while the red text specifies the environmental drivers considered in the model, which includes individual sites. The arrows correspond to the relative influence of environmental variables.

Redundancy analysis identified low variance explained by the effects of time (year of survey) and SSTA at Indonesia (5.9%) and Honduras (4.7%) location. However, when adding site in the RDA model (Figure 5), 69.5% of the variance is explained at Indonesia location while 81.8 is explained at the Honduras location, indicating site variability is the strongest predictor of benthic composition at both these locations.

### 3.3 Change in the benthic community composition from 2012 to 2019

**FIGURE 6:**
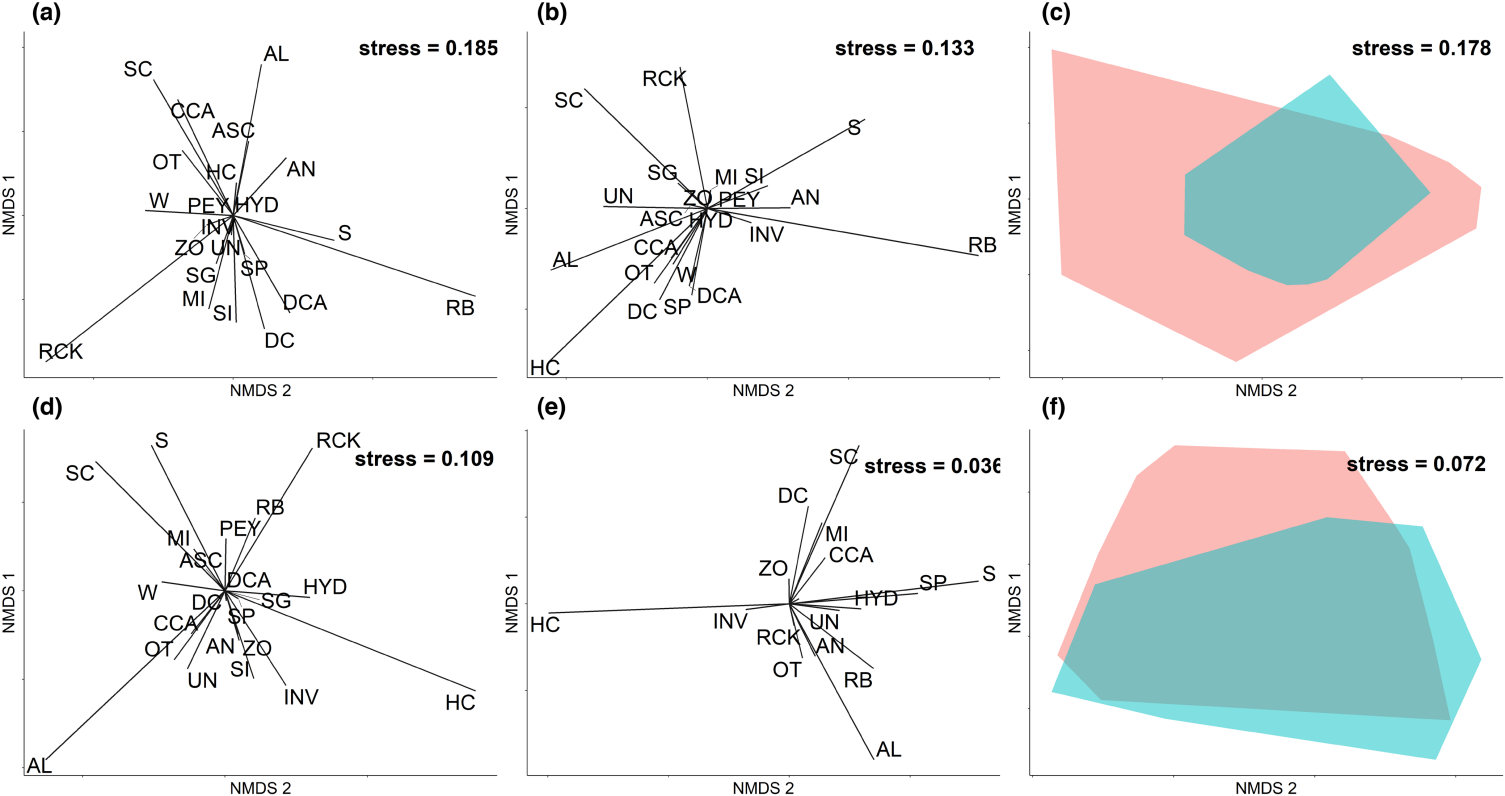
nMDS analysis of the benthic community composition at the Indonesia sites (a–c) and Honduras sites (d–f). (a) and (d) are the composition of individual benthic components from 2012 to 2015. (b) and (e) are response of individual benthic components from 2016 to 2019 (i.e., showing the response of the global marine heatwaves that took place in 2016/2017 [Figure 2]). Letters represent the individual taxa, which are specified in Table 1. (c) and (f) represent the entire ordination space of the benthic composition at each individual reef, where the red polygon bounds the sites from 2012 to 2015, while the blue polygon bound sites from 2016 to 2019.

The relative contribution of individual benthic components to the reef benthic composition is shown from 2012 to 2015 (Figure 6a) and 2016–2019 (Figure 6b), with only slight changes to the benthic components at the Indonesia sites between these time groups. Furthermore, potential simplification/stabilization of the benthic composition at the Indonesia location can be observed based on the entirety of the ordination space occupied pre‐2016 compared with 2016–2019 (Figure 6c). However, at the Honduras location, a drastic change in the benthic components, which drives the community composition, was observed from 2012 to 2015 compared with 2016 to 2019 (Figure 6d,e). This coincided with a shift in the ordination space occupied by each site (Figure 6f).

## 4 DISCUSSION

Our findings reveal dichotomous responses between the two locations of coral reef sites of the Honduras and Indonesia location under SSTAs from 2012 to 2019. Benthic composition varied over time at both locations, but the changes in benthic composition were location specific. Meanwhile, intra‐location variability (i.e., the composition at each site) explained the largest proportion of variance for the benthic composition at both the Indonesia and Honduras locations, indicating fine‐scale variability as a key factor in explaining the benthic composition of these coral reefs.

### 4.1 The relative role of SSTA for driving compositional change

Elevated sea surface temperatures appear to predict coverage of benthic components at the Honduras location only, and not the Indonesia location, indicating that the Honduran reefs surveyed in this study are more susceptible to compositional change under marine heatwaves. This can be seen with the increase in bare rock coverage at the Honduras location and a decrease in sponge coverage in association with SSTAs (Figure 4) but note temporal variations in cover (Figure 3). The increase in bare rock coverage associated with temperature could indicate global warming driving biotic declines of the reef scape through direct and indirect cascading processes (Alvarez‐Filip et al., 2009). Meanwhile, the decrease in sponge coverage identified at the Honduras location is convoluted in the literature. Coral loss attributed to global warming leads to an increase in seaweed abundance, which results in an increased production of dissolved organic carbon that is consumed by sponges. Consequently, nutrients released by sponges enhance seaweed abundance, further inhibiting coral cover (Pawlik et al., 2016). Yet, this process is likely constrained in the long term owing to cascading trophic processes (Lesser & Slattery, 2020). Our findings suggest that this increase will not occur at this location under rising sea temperatures. At the Indonesia location, none of the benthic components were predicted to either increase or decrease from the effect of SSTA, suggesting other factors are driving the benthic composition of these reefs.

In contrast to the effects of SSTAs, temporal patterns of variation predicting the benthic composition of reefs at both the Indonesia and Honduras location are prominent. These temporal patterns, which predict compositional changes at the Indonesia location have been previously recorded for sponges, which showed the strongest temporal increase of all the benthic components. This stark increase is most strongly related to the Sampela site where high sedimentation has driven sponge dominance (Biggerstaff et al., 2017). However, fine‐scale temporal variation in sponge and algae coverage on the coral reefs of the WNP are well documented (Marlow et al., 2020; Rovellini et al., 2019), along with interannual variability of algae coverage (Marlow et al., 2020; Rovellini et al., 2021), which are likely overlooked based on our findings (e.g., Figure 3). The increase in hard corals at the Indonesia location contradicts the assumed temporal stability of hard coral at these reefs (Marlow et al., 2020; Rovellini et al., 2021) but may be a consequence of recovery from a lower baseline because of the 2010 bleaching event (Watt‐Pringle et al., 2022), or a natural cycle where hard corals are increasing owing to temporal variation (Rovellini et al., 2021). This is also indicated by a decrease in coral rubble at the Indonesia location through time, suggesting hard coral cover has displaced dead corals over time. At the Honduras location, increased algae coverage and decreased hard coral cover conform to previous expectation of decline for coral reefs in this region where multiple stressors are compounding coral reef transitions into alternative states (Contreras‐Silva et al., 2020). The decrease in bare rock cover at this location likely relates to the observed increase in algae monopolization and the increase in the soft coral cover through time. Soft corals are taxa assumed to increase on reefs under global warming, as the reduction in hard corals from warming and acidification should allow for soft corals to outcompete hard corals (Inoue et al., 2013), which may be occurring at these Honduran reefs.

### 4.2 Other drivers of reef composition

Given that intrinsic site variability between these two locations appears to be the strongest predictor of benthic composition compared with SSTA and time, it is critical to note other potential drivers of composition at these locations. Firstly, the use of mismatched time series methodology does not capture fine‐scale temporal dynamics of species with faster life histories, such as macroalgae and some sponges (Rovellini et al., 2021). These faster life history traits will also influence rock coverage as a bare substrate will be quickly monopolized by these taxa, yet grazing and/or displacement could occur before sampling between years takes place. However, the general effects of using SSTA over the 52‐week period have been well validated for coral cover (Donovan et al., 2021), which is the most important component of coral reef complexity. The influence of depth was also not considered within our models owing to the dearth of sufficient data. Yet, coral and algae cover at the Honduras location varies by depth (Andradi‐Brown et al., 2016), which is also often assumed to be a refuge for some corals under warming oceans (Bridge et al., 2014). Surveying at both locations encompassed a variety of reef types, zones, and depths, but these data were not specifically recorded during collection so were not included in analyses. However, for corals specifically, depth certainly does not equal refuge, as temperature sensitivity increases with depth (Bongaerts et al., 2017). Furthermore, at the Honduras location, the impacts of grazing herbivores such as *Diadema antillarum*, which support ecosystem function by reducing algae coverage, thus facilitating coral cover increase, were not considered as a driver of benthic composition in this study despite their known positive impacts (Bodmer et al., 2015, 2021). Our analysis also did not consider prevailing ocean currents, such as the influence of the Banda and Flores Sea (Gordon et al., 1994), which at the Indonesia location, is hypothesized to provide cooling waters to corals of the WNP, potentially alleviating bleaching during thermal stress. Finally, the lack of information on the level of coral reef species is also not available from monitoring data, which are likely to be an influential factor for assessing changes to the benthic composition under SSTAs. However, data at this resolution on coral reefs are unlikely feasible with citizen science techniques, therefore a trade‐off between accuracy and resolution must be considered (Done et al., 2017; Gouraguine et al., 2019).

## 5 CONCLUSION

In conclusion, our analyses reveal the composition of reefs at both locations has changed over the last decade, with increasing evidence of changes in Honduras during SSTAs compared with the Indonesia location. At the Indonesia location, temporal variation predicts changes in the benthic composition far more than the effect of elevated sea surface temperatures. However, high variance explained of the benthic composition by adding the site to RDA models indicates other fine‐scale inter‐location factors are likely driving the benthic composition of both these locations. Consequently, continued monitoring of these reefs with a higher taxonomic resolution of data may be beneficial, along with in‐situ temperature recordings. Ultimately, however, the monitoring effort is critical for understanding the local scale composition dynamics of these coral reefs, and how they will change under anthropogenic heating.

## AUTHOR CONTRIBUTIONS

**Jack V. Johnson:** Conceptualization (equal); data curation (equal); formal analysis (equal); investigation (equal); methodology (equal); project administration (equal); resources (equal); software (equal); validation (equal); visualization (equal); writing – original draft (equal); writing – review and editing (equal). **Dan A. Exton:** Conceptualization (equal); methodology (equal); supervision (equal); writing – review and editing (equal). **Jaimie T. A. Dick:** Supervision (equal); writing – review and editing (equal). **Joseph Oakley:** Visualization (equal); writing – review and editing (equal). **Jamaluddin Jompa:** Data curation (equal); project administration (equal); writing – review and editing (equal). **Daniel Pincheira‐Donoso:** Conceptualization (equal); investigation (equal); resources (equal); supervision (equal); validation (equal); writing – original draft (equal); writing – review and editing (equal).

## CONFLICT OF INTEREST

All authors contributed to the manuscript writing and revisions and declare no conflict of interest.

## Supporting information


Figures S1‐S14

## Data Availability

Data are permanently deposited in Dryad (https://doi.org/10.5061/dryad.w3r2280tt) with all code available on our Github page (https://github.com/JackVJohnson/Disparity_between_Indo_Pac_and_Caribbean).

## References

[ece39263-bib-0001] Alvarez‐Filip, L. , Dulvy, N. K. , Gill, J. A. , Côté, I. M. , & Watkinson, A. R. (2009). Flattening of Caribbean coral reefs: Region‐wide declines in architectural complexity. Proceedings of the Royal Society B: Biological Sciences, 276, 3019–3025. 10.1098/rspb.2009.0339 PMC2817220 19515663

[ece39263-bib-0002] Andradi‐Brown, D. A. , Gress, E. , Wright, G. , Exton, D. A. , & Rogers, A. D. (2016). Reef fish community biomass and trophic structure changes across shallow to upper‐mesophotic reefs in the Mesoamerican Barrier Reef, Caribbean. PLoS One, 11, e0156641. 27332811 10.1371/journal.pone.0156641 PMC4917088

[ece39263-bib-0003] Bell, J. J. , Davy, S. K. , Jones, T. , Taylor, M. W. , & Webster, N. S. (2013). Could some coral reefs become sponge reefs as our climate changes? Global Change Biology, 19, 2613–2624. 23553821 10.1111/gcb.12212

[ece39263-bib-0004] Benkwitt, C. E. , Wilson, S. K. , & Graham, N. A. J. (2020). Biodiversity increases ecosystem functions despite multiple stressors on coral reefs. Nature Ecology & Evolution, 4, 919–926. 32424279 10.1038/s41559-020-1203-9

[ece39263-bib-0005] Biggerstaff, A. , Jompa, J. , & Bell, J. J. (2017). Increasing benthic dominance of the phototrophic sponge Lamellodysidea herbacea on a sedimented reef within the Coral Triangle. Marine Biology, 164, 220.

[ece39263-bib-0006] Bodmer, M. D. V. , Rogers, A. D. , Speight, M. R. , Lubbock, N. , & Exton, D. A. (2015). Using an isolated population boom to explore barriers to recovery in the keystone Caribbean coral reef herbivore *Diadema antillarum* . Coral Reefs, 34, 1011–1021.

[ece39263-bib-0007] Bodmer, M. D. V. , Wheeler, P. M. , Anand, P. , Cameron, S. E. , Hintikka, S. , Cai, W. , Borcsok, A. O. , & Exton, D. A. (2021). The ecological importance of habitat complexity to the Caribbean coral reef herbivore *Diadema antillarum*: Three lines of evidence. Scientific Reports, 11, 9382. 33931650 10.1038/s41598-021-87232-9 PMC8087687

[ece39263-bib-0008] Boilard, A. , Dubé, C. E. , Gruet, C. , Mercière, A. , Hernandez‐Agreda, A. , & Derome, N. (2020). Defining coral bleaching as a microbial dysbiosis within the coral holobiont. Microorganisms, 8, 1682. 10.3390/microorganisms8111682 PMC7692791 33138319

[ece39263-bib-0009] Bongaerts, P. , Riginos, C. , Brunner, R. , Englebert, N. , Smith, S. R. , & Hoegh‐Guldberg, O. (2017). Deep reefs are not universal refuges: Reseeding potential varies among coral species. Science Advances, 3, e1602373. 28246645 10.1126/sciadv.1602373 PMC5310828

[ece39263-bib-0010] Bozec, Y.‐M. , Doropoulos, C. , Roff, G. , & Mumby, P. J. (2019). Transient grazing and the dynamics of an unanticipated coral–algal phase shift. Ecosystems, 22, 296–311.

[ece39263-bib-0011] Brandl, S. J. , Rasher, D. B. , Côté, I. M. , Casey, J. M. , Darling, E. S. , Lefcheck, J. S. , & Duffy, J. E. (2019). Coral reef ecosystem functioning: Eight core processes and the role of biodiversity. Frontiers in Ecology and the Environment, 17, 445–454.

[ece39263-bib-0012] Bridge, T. C. L. , Hoey, A. S. , Campbell, S. J. , Muttaqin, E. , Rudi, E. , Fadli, N. , & Baird, A. H. (2014). Depth‐dependent mortality of reef corals following a severe bleaching event: Implications for thermal refuges and population recovery. F1000Research, 2, 187. 10.12688/f1000research.2-187.v1 PMC3938179 24627789

[ece39263-bib-0013] Bruno, J. F. , & Selig, E. R. (2007). Regional decline of coral cover in the indo‐pacific: Timing, extent, and subregional comparisons. PLoS One, 2, e711. 17684557 10.1371/journal.pone.0000711 PMC1933595

[ece39263-bib-0014] Bürkner, P.‐C. (2017). brms: An R package for Bayesian multilevel models using stan. Journal of Statistical Software, 80, 1–28.

[ece39263-bib-0015] Carpenter, B. , Gelman, A. , Hoffman, M. D. , Lee, D. , Goodrich, B. , Betancourt, M. , Brubaker, M. , Guo, J. , Li, P. , & Riddell, A. (2017). Stan: A probabilistic programming language. Journal of Statistical Software, 76, 1–32. 10.18637/jss.v076.i01 PMC9788645 36568334

[ece39263-bib-0016] Clifton, J. , Unsworth, R. K. , & Smith, D. J. (2010). Marine research and conservation in the coral triangle. Nova Science Publishers.

[ece39263-bib-0017] Contreras‐Silva, A. I. , Tilstra, A. , Migani, V. , Thiel, A. , Pérez‐Cervantes, E. , Estrada‐Saldívar, N. , Elias‐Ilosvay, X. , Mott, C. , Alvarez‐Filip, L. , & Wild, C. (2020). A meta‐analysis to assess long‐term spatiotemporal changes of benthic coral and macroalgae cover in the Mexican Caribbean. Scientific Reports, 10, 8897. 32483234 10.1038/s41598-020-65801-8 PMC7264131

[ece39263-bib-0018] Crabbe, J. M. , & Smith, D. J. (2002). Comparison of two reef sites in the Wakatobi Marine National Park (SE Sulawesi, Indonesia) using digital image analysis. Coral Reefs, 21, 242–244.

[ece39263-bib-0019] Cullen, L. C. , Pretty, J. , Smith, D. , & Pilgrim, S. E. (2007). Links between local ecological knowledge and wealth in indigenous communities of Indonesia: Implications for conservation of marine resources. The International Journal of Interdisciplinary Social Sciences, 2, 289–299.

[ece39263-bib-0020] Done, T. , Roelfsema, C. , Harvey, A. , Schuller, L. , Hill, J. , Schläppy, M.‐L. , Lea, A. , Bauer‐Civiello, A. , & Loder, J. (2017). Reliability and utility of citizen science reef monitoring data collected by Reef Check Australia, 2002–2015. Marine Pollution Bulletin, 117, 148–155. 28162251 10.1016/j.marpolbul.2017.01.054

[ece39263-bib-0021] Donovan, M. K. , Burkepile, D. E. , Kratochwill, C. , Shlesinger, T. , Sully, S. , Oliver, T. A. , Hodgson, G. , Freiwald, J. , & van Woesik, R. (2021). Local conditions magnify coral loss after marine heatwaves. Science, 372, 977–980. 34045353 10.1126/science.abd9464

[ece39263-bib-0022] Douglas, A. E. (2003). Coral bleaching––How and why? Marine Pollution Bulletin, 46, 385–392. 12705909 10.1016/S0025-326X(03)00037-7

[ece39263-bib-0023] Exton, D. A. , Ahmadia, G. N. , Cullen‐Unsworth, L. C. , Jompa, J. , May, D. , Rice, J. , Simonin, P. W. , Unsworth, R. K. F. , & Smith, D. J. (2019). Artisanal fish fences pose broad and unexpected threats to the tropical coastal seascape. Nature Communications, 10, 2100. 10.1038/s41467-019-10051-0 PMC6529422 31113956

[ece39263-bib-0024] Ferguson, J. M. , Reichert, B. E. , Fletcher, R. J., Jr. , & Jager, H. I. (2017). Detecting population–environmental interactions with mismatched time series data. Ecology, 98, 2813–2822. 28759123 10.1002/ecy.1966 PMC5704982

[ece39263-bib-0025] Fisher, R. , O'Leary, R. A. , Low‐Choy, S. , Mengersen, K. , Knowlton, N. , Brainard, R. E. , & Caley, M. J. (2015). Species richness on coral reefs and the pursuit of convergent global estimates. Current Biology, 25, 500–505. 25639239 10.1016/j.cub.2014.12.022

[ece39263-bib-0026] Fitt, W. K. , Brown, B. E. , Warner, M. E. , & Dunne, R. P. (2001). Coral bleaching: Interpretation of thermal tolerance limits and thermal thresholds in tropical corals. Coral Reefs, 20, 51–65.

[ece39263-bib-0027] Foale, S. , Adhuri, D. , Aliño, P. , Allison, E. H. , Andrew, N. , Cohen, P. , Evans, L. , Fabinyi, M. , Fidelman, P. , Gregory, C. , Stacey, N. , Tanzer, J. , & Weeratunge, N. (2013). Food security and the Coral Triangle Initiative. Marine Policy, 38, 174–183.

[ece39263-bib-0028] Friedlander, A. M. , & Parrish, J. D. (1998). Habitat characteristics affecting fish assemblages on a Hawaiian coral reef. Journal of Experimental Marine Biology and Ecology, 224, 1–30.

[ece39263-bib-0029] Fulton, C. J. , Abesamis, R. A. , Berkström, C. , Depczynski, M. , Graham, N. A. J. , Holmes, T. H. , Kulbicki, M. , Noble, M. M. , Radford, B. T. , Tano, S. , Tinkler, P. , Wernberg, T. , & Wilson, S. K. (2019). Form and function of tropical macroalgal reefs in the Anthropocene. Functional Ecology, 33, 989–999.

[ece39263-bib-0030] Gordon, A. L. , Ffield, A. , & Ilahude, A. G. (1994). Thermocline of the Flores and Banda seas. Journal of Geophysical Research: Oceans, 99, 18235–18242.

[ece39263-bib-0031] Gouraguine, A. , Moranta, J. , Ruiz‐Frau, A. , Hinz, H. , Reñones, O. , Ferse, S. C. A. , Jompa, J. , & Smith, D. J. (2019). Citizen science in data and resource‐limited areas: A tool to detect long‐term ecosystem changes. PLoS One, 14, e0210007. 30625207 10.1371/journal.pone.0210007 PMC6326458

[ece39263-bib-0032] Graham, N. A. J. , Bellwood, D. R. , Cinner, J. E. , Hughes, T. P. , Norström, A. V. , & Nyström, M. (2013). Managing resilience to reverse phase shifts in coral reefs. Frontiers in Ecology and the Environment, 11, 541–548.

[ece39263-bib-0033] Harrison, H. B. , Álvarez‐Noriega, M. , Baird, A. H. , Heron, S. F. , MacDonald, C. , & Hughes, T. P. (2019). Back‐to‐back coral bleaching events on isolated atolls in the Coral Sea. Coral Reefs, 38, 713–719.

[ece39263-bib-0034] Hughes, T. P. , Anderson, K. D. , Connolly, S. R. , Heron, S. F. , Kerry, J. T. , Lough, J. M. , Baird, A. H. , Baum, J. K. , Berumen, M. L. , Bridge, T. C. , Claar, D. C. , Eakin, C. M. , Gilmour, J. P. , Graham, N. A. J. , Harrison, H. , Hobbs, J.‐P. A. , Hoey, A. S. , Hoogenboom, M. , Lowe, R. J. , … Wilson, S. K. (2018). Spatial and temporal patterns of mass bleaching of corals in the Anthropocene. Science, 359, 80–83. 29302011 10.1126/science.aan8048

[ece39263-bib-0035] Hughes, T. P. , Barnes, M. L. , Bellwood, D. R. , Cinner, J. E. , Cumming, G. S. , Jackson, J. B. C. , Kleypas, J. , van de Leemput, I. A. , Lough, J. M. , Morrison, T. H. , Palumbi, S. R. , van Nes, E. H. , & Scheffer, M. (2017). Coral reefs in the Anthropocene. Nature, 546, 82–90. 28569801 10.1038/nature22901

[ece39263-bib-0036] Hughes, T. P. , Kerry, J. T. , Baird, A. H. , Connolly, S. R. , Chase, T. J. , Dietzel, A. , Hill, T. , Hoey, A. S. , Hoogenboom, M. O. , Jacobson, M. , Kerswell, A. , Madin, J. S. , Mieog, A. , Paley, A. S. , Pratchett, M. S. , Torda, G. , & Woods, R. M. (2019). Global warming impairs stock–recruitment dynamics of corals. Nature, 568, 387–390. 30944475 10.1038/s41586-019-1081-y

[ece39263-bib-0037] Hughes, T. P. , Kerry, J. T. , Baird, A. H. , Connolly, S. R. , Dietzel, A. , Eakin, C. M. , Heron, S. F. , Hoey, A. S. , Hoogenboom, M. O. , Liu, G. , McWilliam, M. J. , Pears, R. J. , Pratchett, M. S. , Skirving, W. J. , Stella, J. S. , & Torda, G. (2018). Global warming transforms coral reef assemblages. Nature, 556, 492–496. 29670282 10.1038/s41586-018-0041-2

[ece39263-bib-0038] Hughes, T. P. , Rodrigues, M. J. , Bellwood, D. R. , Ceccarelli, D. , Hoegh‐Guldberg, O. , McCook, L. , Moltschaniwskyj, N. , Pratchett, M. S. , Steneck, R. S. , & Willis, B. (2007). Phase shifts, herbivory, and the resilience of coral reefs to climate change. Current Biology, 17, 360–365. 17291763 10.1016/j.cub.2006.12.049

[ece39263-bib-0039] Inoue, S. , Kayanne, H. , Yamamoto, S. , & Kurihara, H. (2013). Spatial community shift from hard to soft corals in acidified water. Nature Clim Change, 3, 683–687.

[ece39263-bib-0040] Johnson, J. V. , Dick, J. T. A. , & Pincheira‐Donoso, D. (2022a). Local anthropogenic stress does not exacerbate coral bleaching under global climate change. Global Ecology and Biogeography, 31, 1228–1236.

[ece39263-bib-0041] Johnson, J. V. , Dick, J. T. A. , & Pincheira‐Donoso, D. (2022b). Marine protected areas do not buffer corals from bleaching under global warming. BMC Ecology and Evolution, 22, 58. 35508975 10.1186/s12862-022-02011-y PMC9066861

[ece39263-bib-0042] Ledlie, M. H. , Graham, N. A. J. , Bythell, J. C. , Wilson, S. K. , Jennings, S. , Polunin, N. V. C. , & Hardcastle, J. (2007). Phase shifts and the role of herbivory in the resilience of coral reefs. Coral Reefs, 26, 641–653.

[ece39263-bib-0043] Lesser, M. P. , & Slattery, M. (2020). Will coral reef sponges be winners in the Anthropocene? Global Change Biology, 26, 3202–3211. 32052520 10.1111/gcb.15039

[ece39263-bib-0044] Liu, G. , Heron, S. F. , Eakin, C. M. , Muller‐Karger, F. E. , Vega‐Rodriguez, M. , Guild, L. S. , De La Cour, J. L. , Geiger, E. F. , Skirving, W. J. , Burgess, T. F. R. , Strong, A. E. , Harris, A. , Maturi, E. , Ignatov, A. , Sapper, J. , Li, J. , & Lynds, S. (2014). Reef‐scale thermal stress monitoring of coral ecosystems: New 5‐km global products from NOAA Coral Reef Watch. Remote Sensing, 6, 11579–11606.

[ece39263-bib-0045] Marlow, J. , Haris, A. , Jompa, J. , Werorilangi, S. , Bates, T. , Bennett, H. , & Bell, J. J. (2020). Spatial variation in the benthic community composition of coral reefs in the Wakatobi Marine National Park, Indonesia: Updated baselines and limited benthic community shifts. Journal of the Marine Biological Association of the United Kingdom, 100, 37–44.

[ece39263-bib-0046] Marlow, J. , Smith, D. , Werorilang, S. , & Bell, J. (2018). Sedimentation limits the erosion rate of a bioeroding sponge. Marine Ecology, 39, e12483.

[ece39263-bib-0047] McClanahan, T. R. , Darling, E. S. , Maina, J. M. , Muthiga, N. A. , Agata, S. D. , Jupiter, S. D. , Arthur, R. , Wilson, S. K. , Mangubhai, S. , Nand, Y. , Ussi, A. M. , Humphries, A. T. , Patankar, V. J. , MMM, G. , Keith, S. A. , Shedrawi, G. , Julius, P. , Grimsditch, G. , Ndagala, J. , & Leblond, J. (2019). Temperature patterns and mechanisms influencing coral bleaching during the 2016 El Niño. Nature Climate Change, 9, 845–851.

[ece39263-bib-0048] McClanahan, T. R. , Maina, J. M. , Darling, E. S. , Guillaume, M. M. M. , Muthiga, N. A. , D'agata, S. , Leblond, J. , Arthur, R. , Jupiter, S. D. , Wilson, S. K. , Mangubhai, S. , Ussi, A. M. , Humphries, A. T. , Patankar, V. , Shedrawi, G. , Julius, P. , Ndagala, J. , & Grimsditch, G. (2020). Large geographic variability in the resistance of corals to thermal stress. Global Ecology and Biogeography, 29, 2229–2247.

[ece39263-bib-0049] Moberg, F. , & Folke, C. (1999). Ecological goods and services of coral reef ecosystems. Ecological Economics, 29, 215–233.

[ece39263-bib-0050] Norström, A. V. , Nyström, M. , Jouffray, J.‐B. , Folke, C. , Graham, N. A. , Moberg, F. , Olsson, P. , & Williams, G. J. (2016). Guiding coral reef futures in the Anthropocene. Frontiers in Ecology and the Environment, 14, 490–498.

[ece39263-bib-0051] Oksanen, J. , Blanchet, F. G. , Kindt, R. , Legendre, P. , Minchin, P. R. , O'hara, R. , Simpson, G. L. , Solymos, P. , Stevens, M. H. H. , & Wagner, H. (2013). Package ‘vegan.’ Community ecology package, version 2:1–295.

[ece39263-bib-0052] Oliver, T. H. , Heard, M. S. , Isaac, N. J. B. , Roy, D. B. , Procter, D. , Eigenbrod, F. , Freckleton, R. , Hector, A. , Orme, C. D. L. , Petchey, O. L. , Proença, V. , Raffaelli, D. , Suttle, K. B. , Mace, G. M. , Martín‐López, B. , Woodcock, B. A. , & Bullock, J. M. (2015). Biodiversity and resilience of ecosystem functions. Trends in Ecology & Evolution, 30, 673–684. 26437633 10.1016/j.tree.2015.08.009

[ece39263-bib-0053] Pawlik, J. R. , Burkepile, D. E. , & Thurber, R. V. (2016). A vicious circle? Altered carbon and nutrient cycling may explain the low resilience of caribbean coral reefs. Bioscience, 66, 470–476.

[ece39263-bib-0054] Powell, A. , Smith, D. J. , Hepburn, L. J. , Jones, T. , Berman, J. , Jompa, J. , & Bell, J. J. (2014). Reduced diversity and high sponge abundance on a sedimented indo‐pacific reef system: implications for future changes in environmental quality. PLoS One, 9, e85253. 24475041 10.1371/journal.pone.0085253 PMC3901660

[ece39263-bib-0055] Pratchett, M. S. , Hoey, A. S. , Wilson, S. K. , Messmer, V. , & Graham, N. A. J. (2011). Changes in biodiversity and functioning of reef fish assemblages following coral bleaching and coral loss. Diversity, 3, 424–452.

[ece39263-bib-0056] R Core Team . (2021). R: A language and environment for statistical computing. R Foundation for Statistical Computing.

[ece39263-bib-0057] Roff, G. , & Mumby, P. J. (2012). Global disparity in the resilience of coral reefs. Trends in Ecology & Evolution, 27, 404–413. 22658876 10.1016/j.tree.2012.04.007

[ece39263-bib-0058] Rovellini, A. , Dunn, M. R. , Fulton, E. A. , Webster, N. S. , Smith, D. J. , Jompa, J. , Haris, A. , Berman, J. , & Bell, J. J. (2019). Decadal variability in sponge abundance and biodiversity on an Indo‐Pacific coral reef. Marine Ecology Progress Series, 620, 63–76.

[ece39263-bib-0059] Rovellini, A. , Dunn, M. R. , Fulton, E. A. , Woods, L. , Jompa, J. , Haris, A. , & Bell, J. J. (2021). Interannual variability and decadal stability of benthic organisms on an Indonesian coral reef. Journal of the Marine Biological Association of the United Kingdom, 101, 221–231.

[ece39263-bib-0060] Schiettekatte, N. M. D. , Brandl, S. J. , & Casey, J. M. (2022). fishualize: Color palettes based on fish species.

[ece39263-bib-0061] Suggett, D. J. , & Smith, D. J. (2020). Coral bleaching patterns are the outcome of complex biological and environmental networking. Global Change Biology, 26, 68–79. 31618499 10.1111/gcb.14871

[ece39263-bib-0062] Sully, S. , Burkepile, D. E. , Donovan, M. K. , Hodgson, G. , & van Woesik, R. (2019). A global analysis of coral bleaching over the past two decades. Nature Communications, 10, 1264. 10.1038/s41467-019-09238-2 PMC6427037 30894534

[ece39263-bib-0063] Titus, B. M. , Daly, M. , & Exton, D. A. (2015). Do reef fish habituate to diver presence? Evidence from two reef sites with contrasting historical levels of SCUBA Intensity in the Bay Islands, Honduras. PLoS One, 10, e0119645. 25807543 10.1371/journal.pone.0119645 PMC4373863

[ece39263-bib-0064] Warner, M. E. , Fitt, W. K. , & Schmidt, G. W. (1999). Damage to photosystem II in symbiotic dinoflagellates: A determinant of coral bleaching. PNAS, 96, 8007–8012. 10393938 10.1073/pnas.96.14.8007 PMC22178

[ece39263-bib-0065] Watt‐Pringle, R. , Smith, D. J. , Ambo‐Rappe, R. , Lamont, T. A. C. , & Jompa, J. (2022). Suppressed recovery of functionally important branching Acropora drives coral community composition changes following mass bleaching in Indonesia. Coral Reefs. 10.1007/s00338-022-02275-2

[ece39263-bib-0066] Woodhead, A. J. , Hicks, C. C. , Norström, A. V. , Williams, G. J. , & Graham, N. A. J. (2019). Coral reef ecosystem services in the Anthropocene. Functional Ecology, 33, 1023–1034.

