# Supplementary material for: The relative influence of sea surface temperature anomalies on the benthic composition of an Indo‐Pacific and Caribbean coral reef over the last decade
Source: Ecol Evol. 2022 Sep 6;12(9):10.1002/ece3.9263. doi: 10.1002/ece3.9263 (PMC9448965; doi:10.1002/ece3.9263)
Supplement: Supplementary file 1 — Figures S1‐S14 [file ECE3-12--s001.docx]

**Supplementary material**


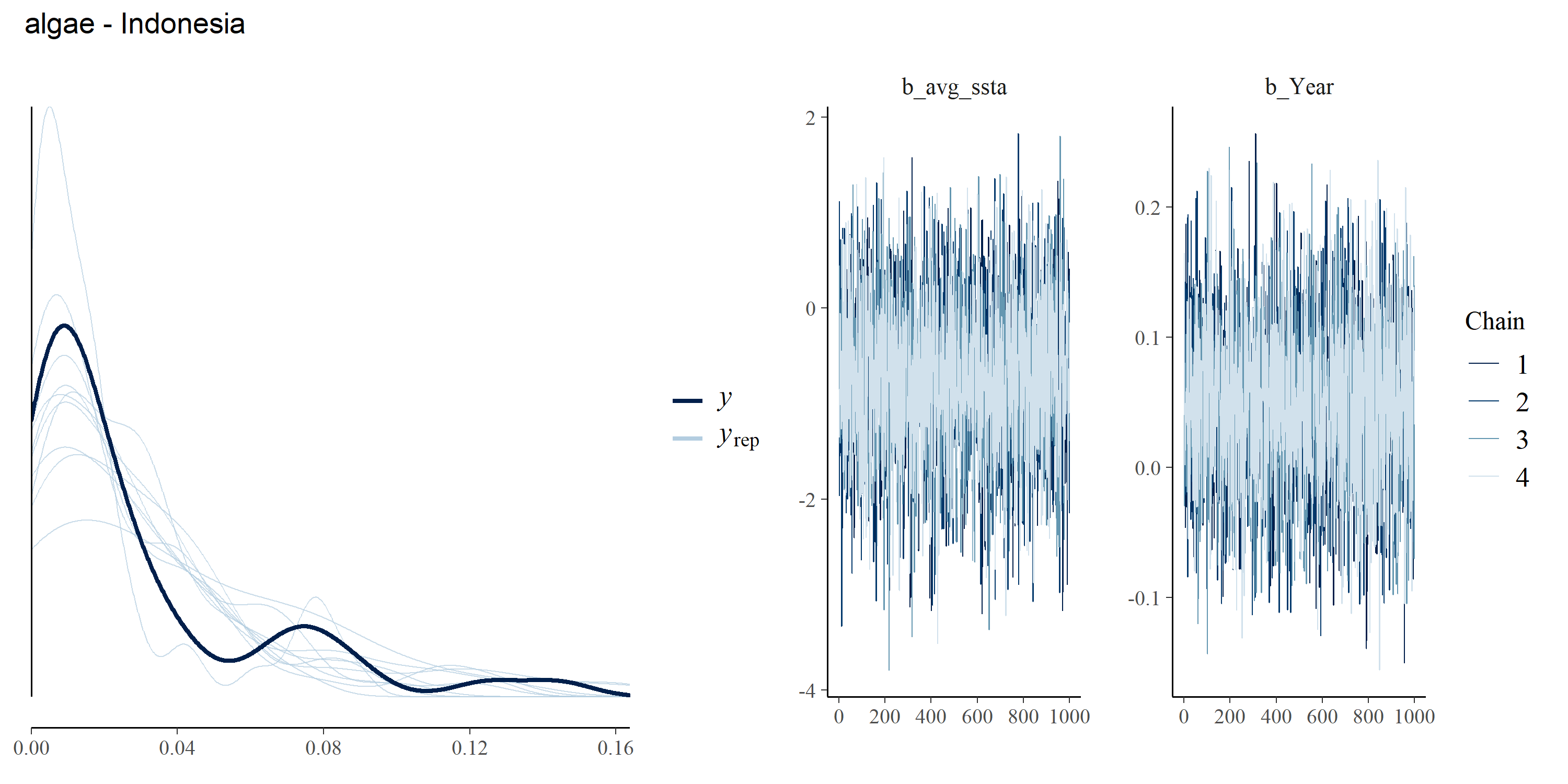


Fig S1. Posterior predictive check and trace plot for Algae coverage, Indonesia


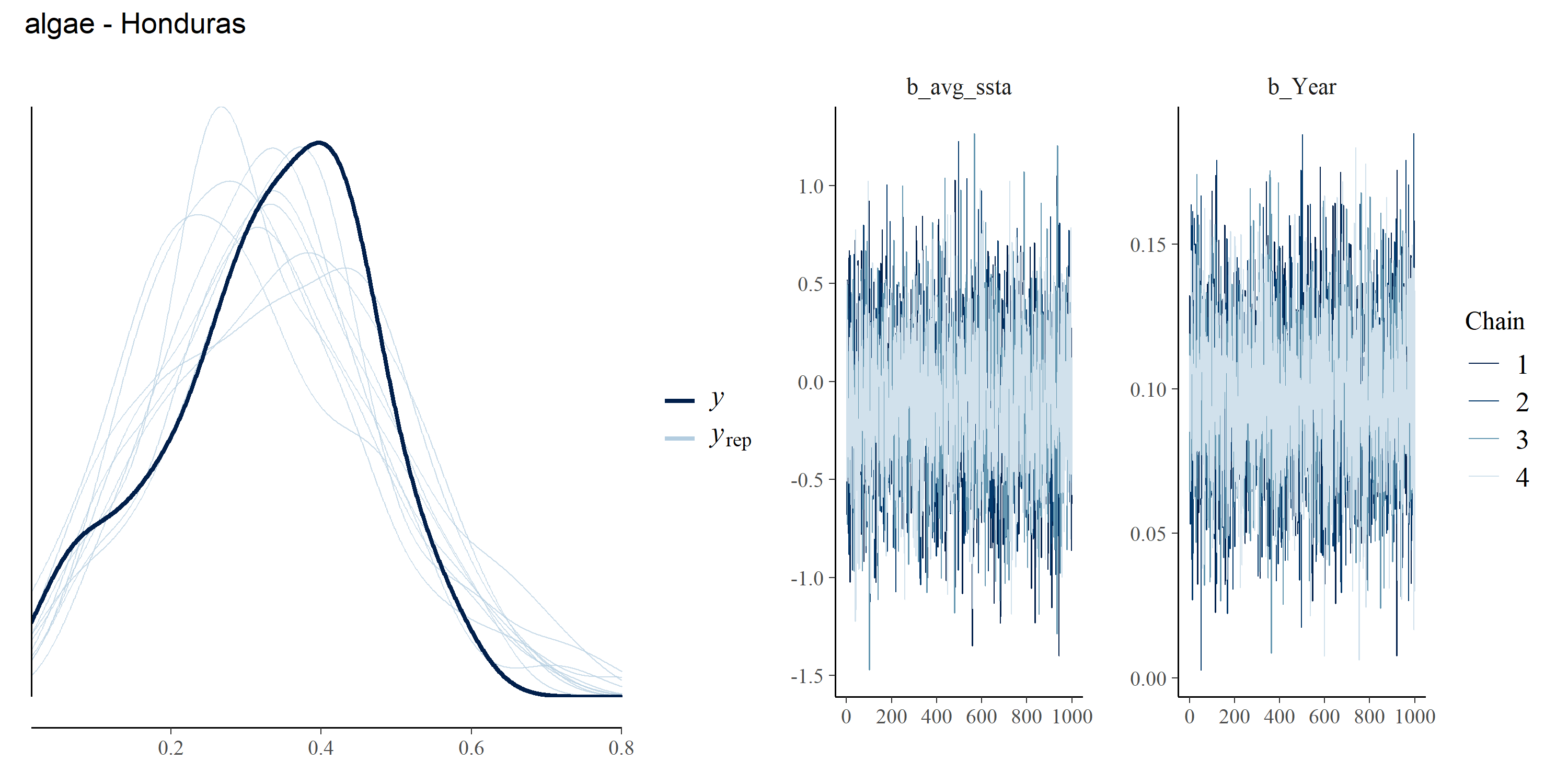


Fig S2. Posterior predictive check and trace plot for Algae coverage, Honduras


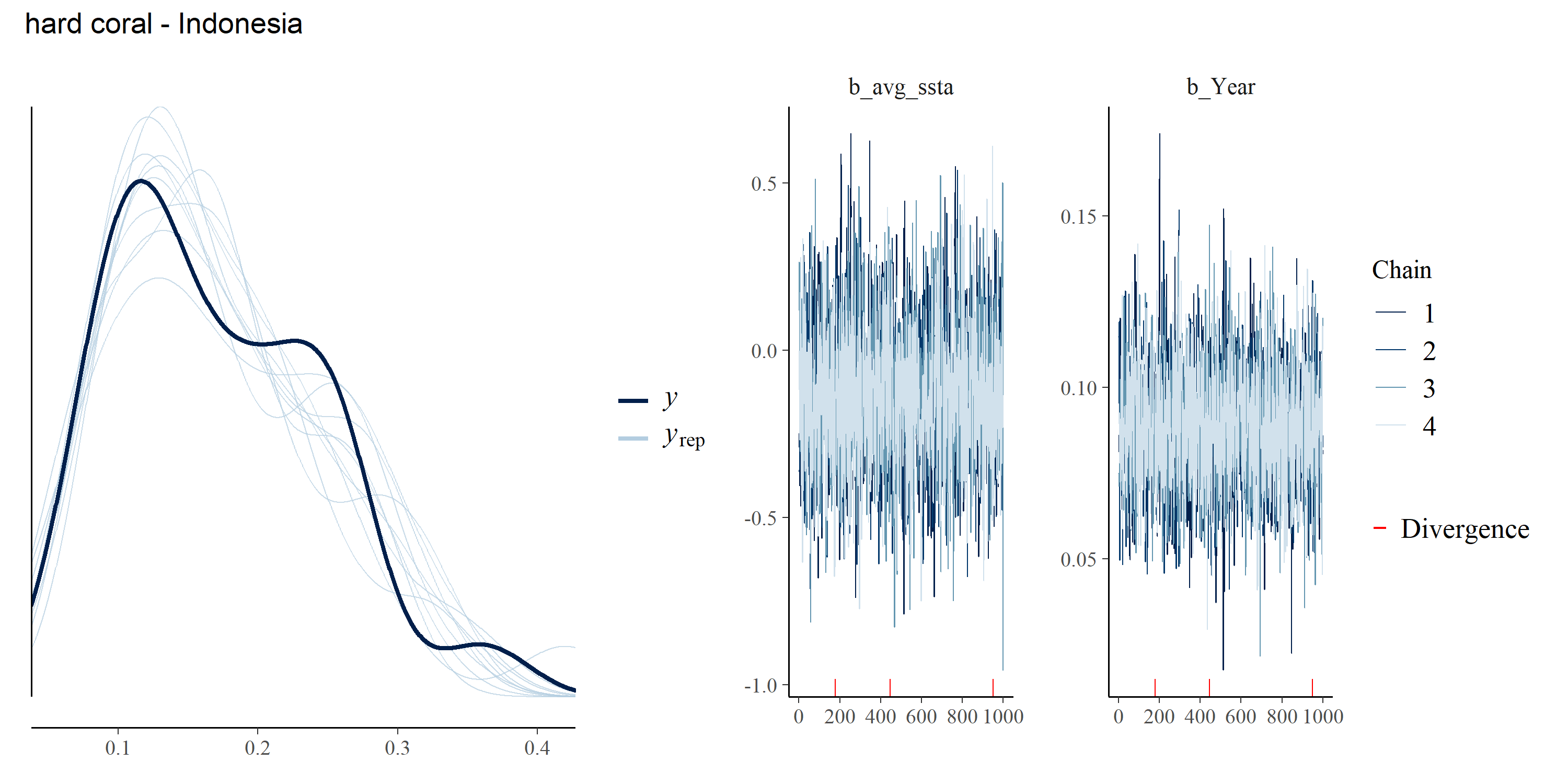


Fig S3. Posterior predictive check and trace plot for hard coral coverage, Indonesia


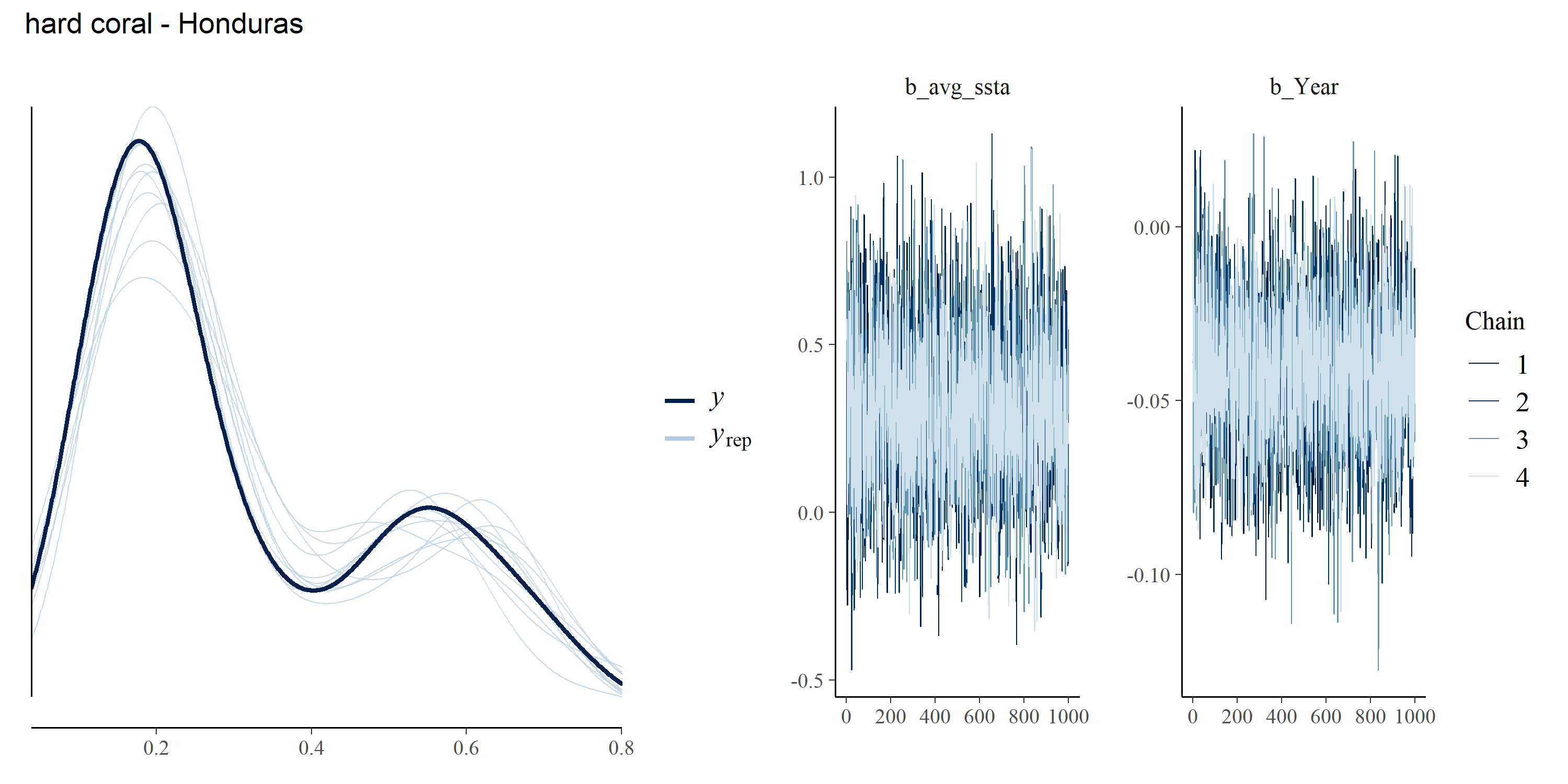


Fig S4. Posterior predictive check and trace plot for hard coral coverage, Honduras


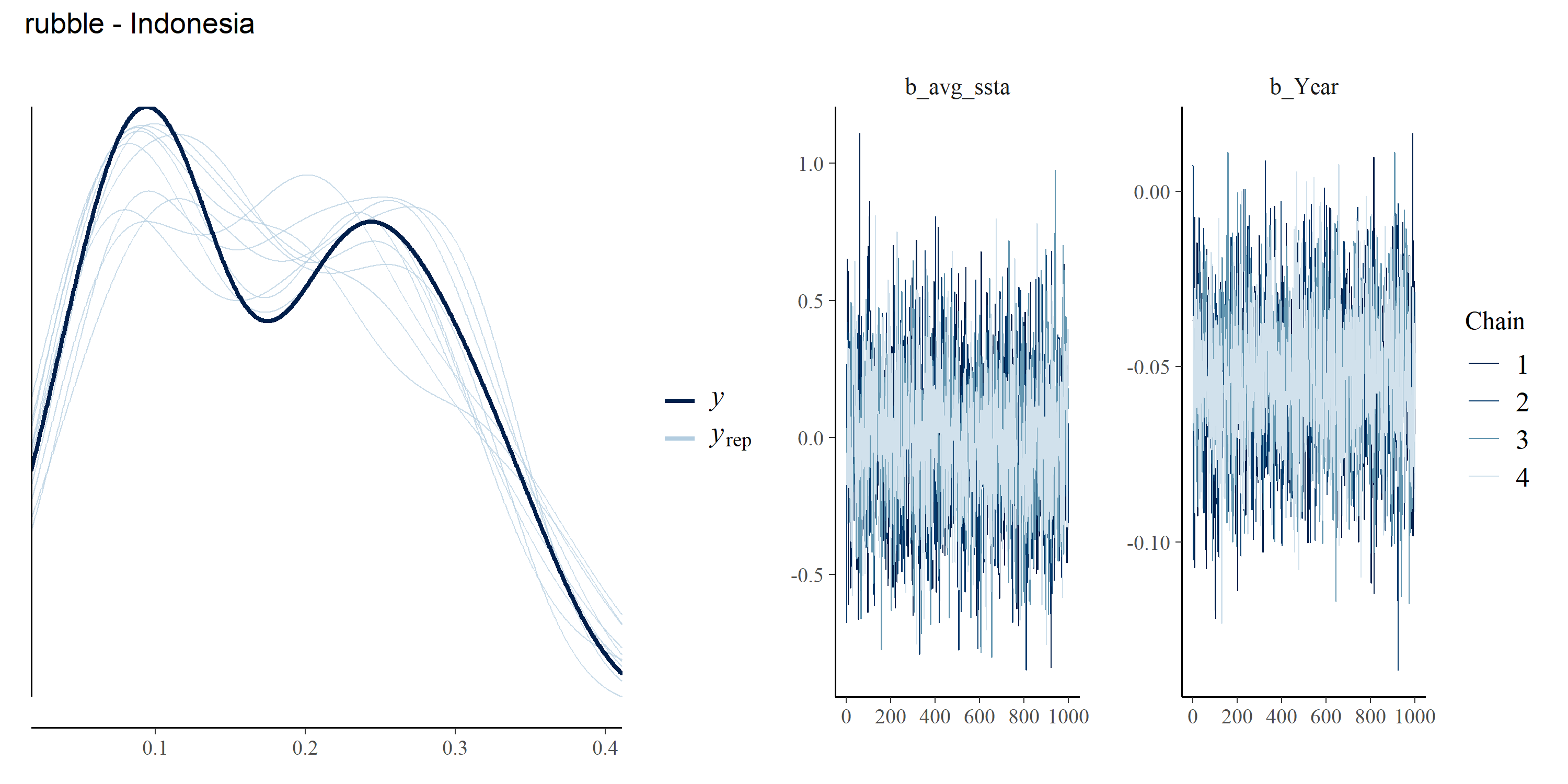


Fig S5. Posterior predictive check and trace plot for rubble coverage, Indonesia


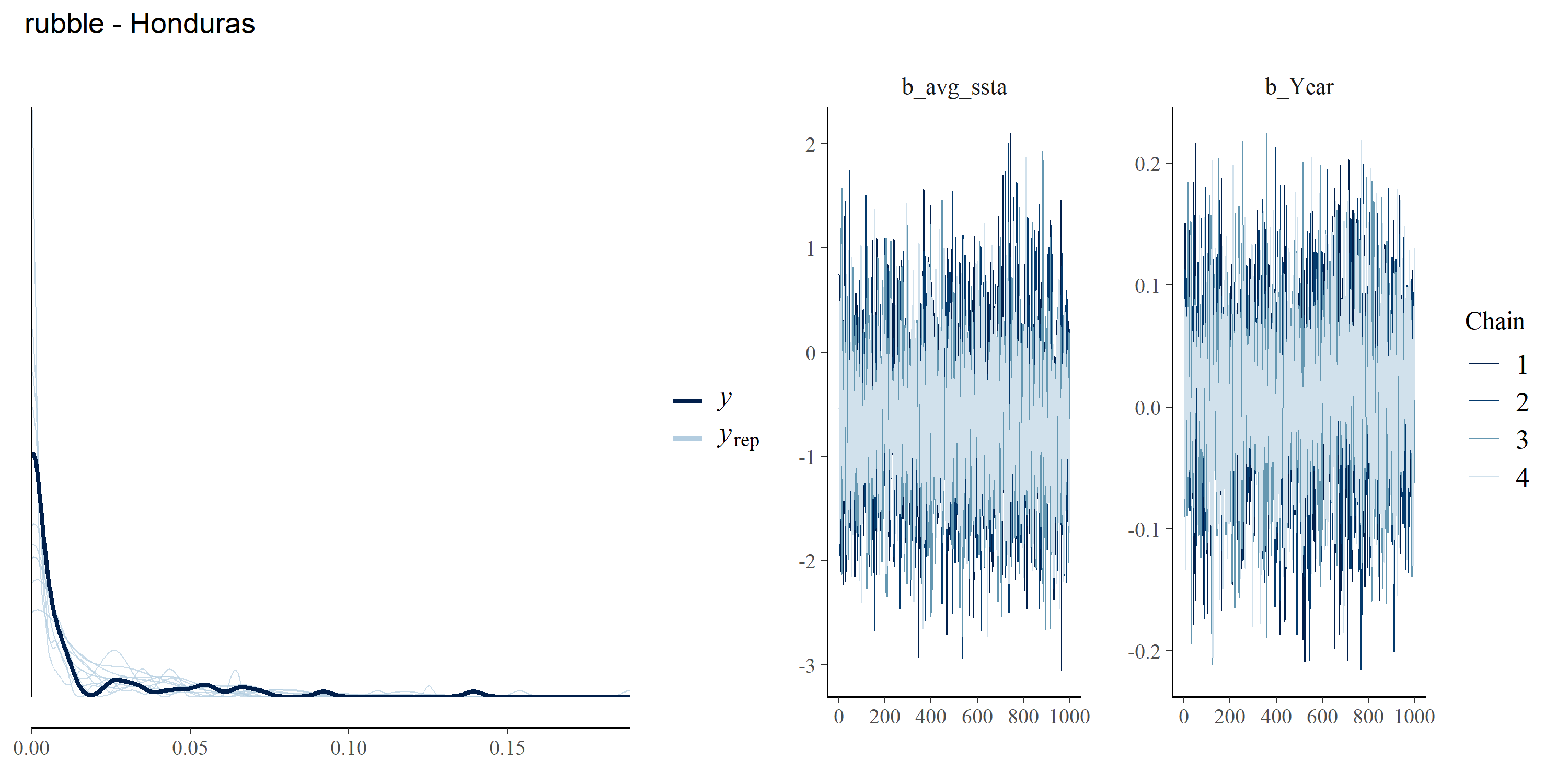


Fig S6. Posterior predictive check and trace plot for rubble coverage, Honduras


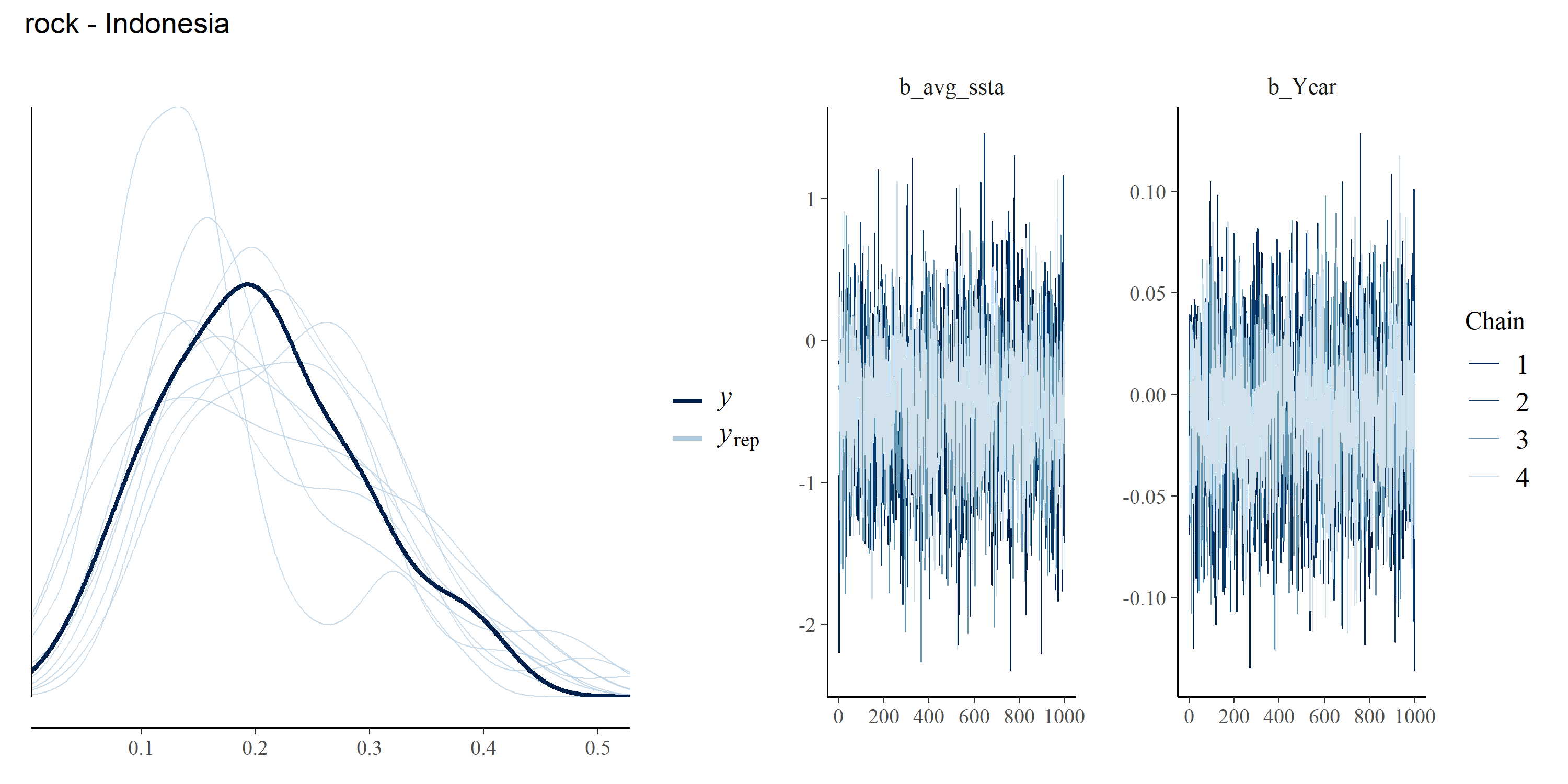


Fig S7. Posterior predictive check and trace plot for rock coverage, Indonesia


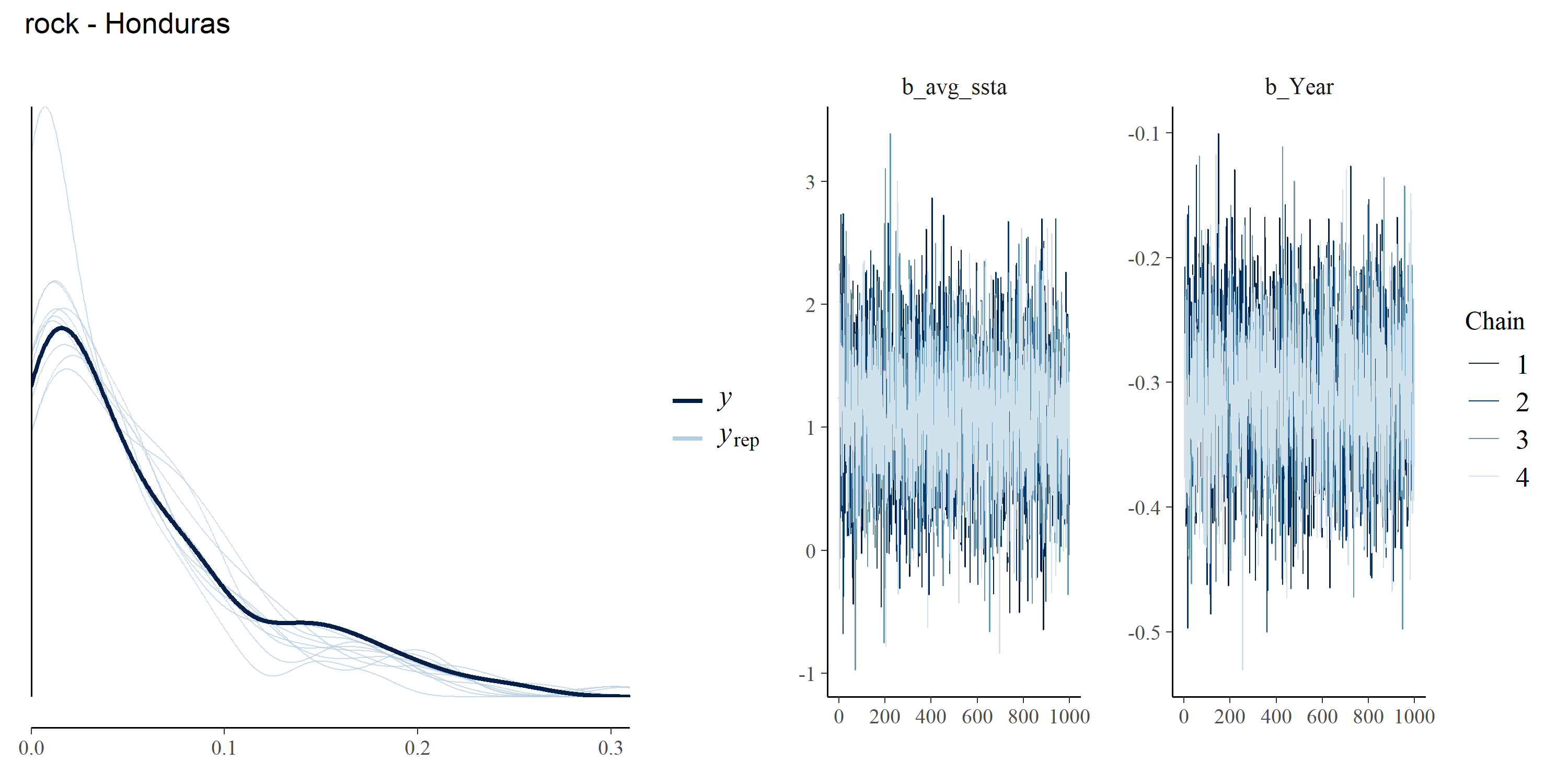


Fig S8. Posterior predictive check and trace plot for rock coverage, Honduras


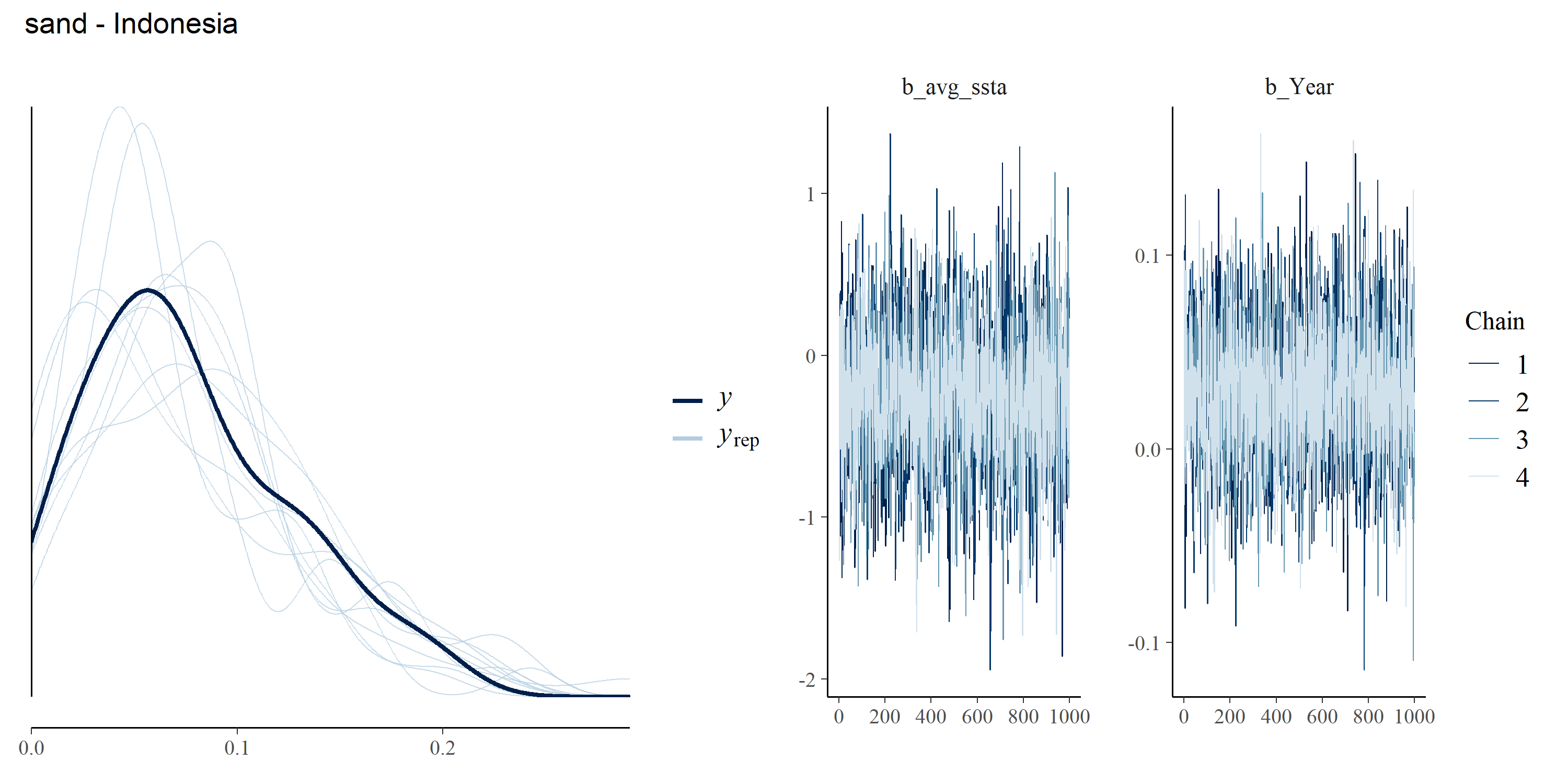


Fig S9. Posterior predictive check and trace plot for sand coverage, Indonesia


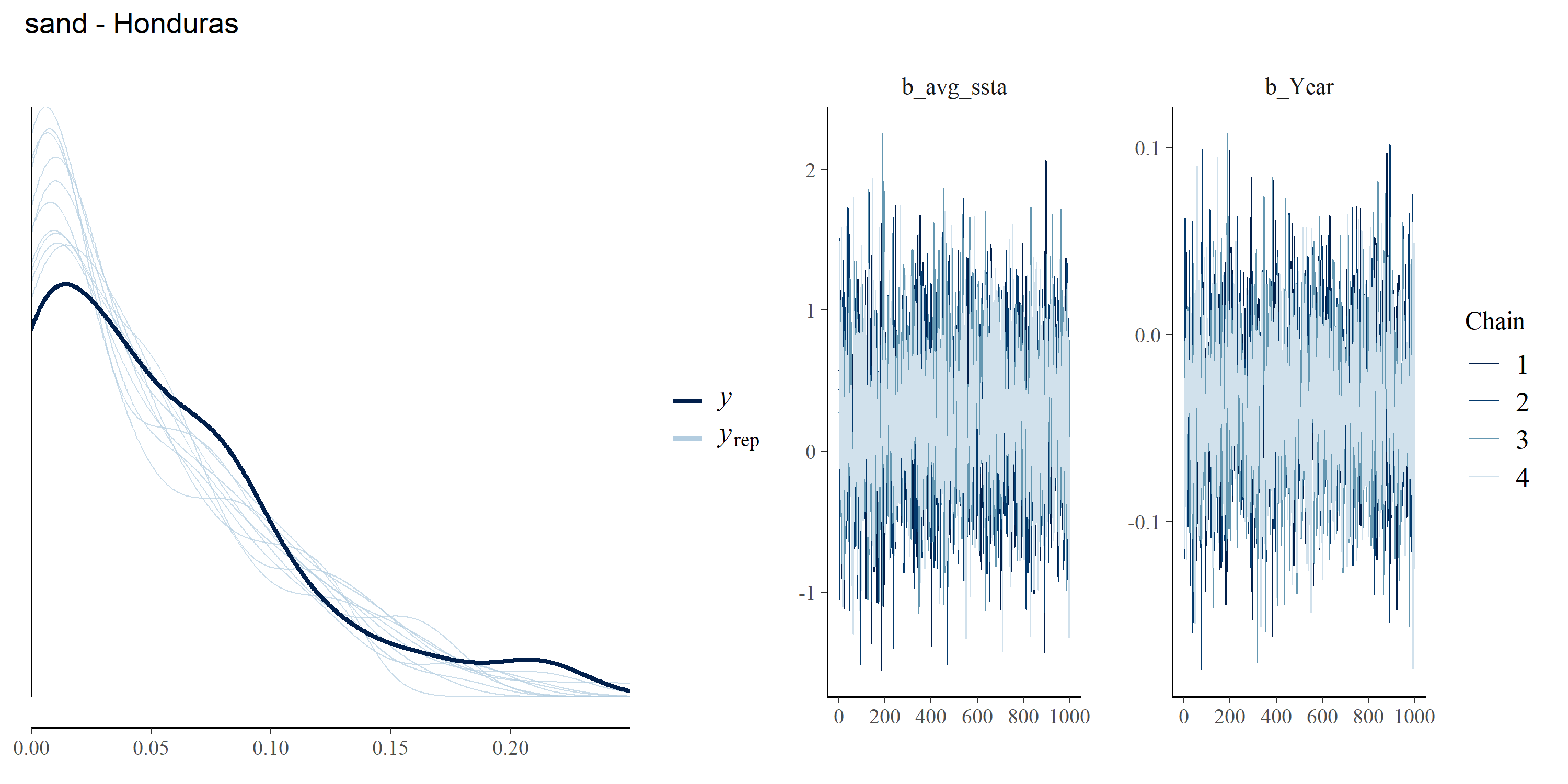


Fig S10. Posterior predictive check and trace plot for sand coverage, Honduras


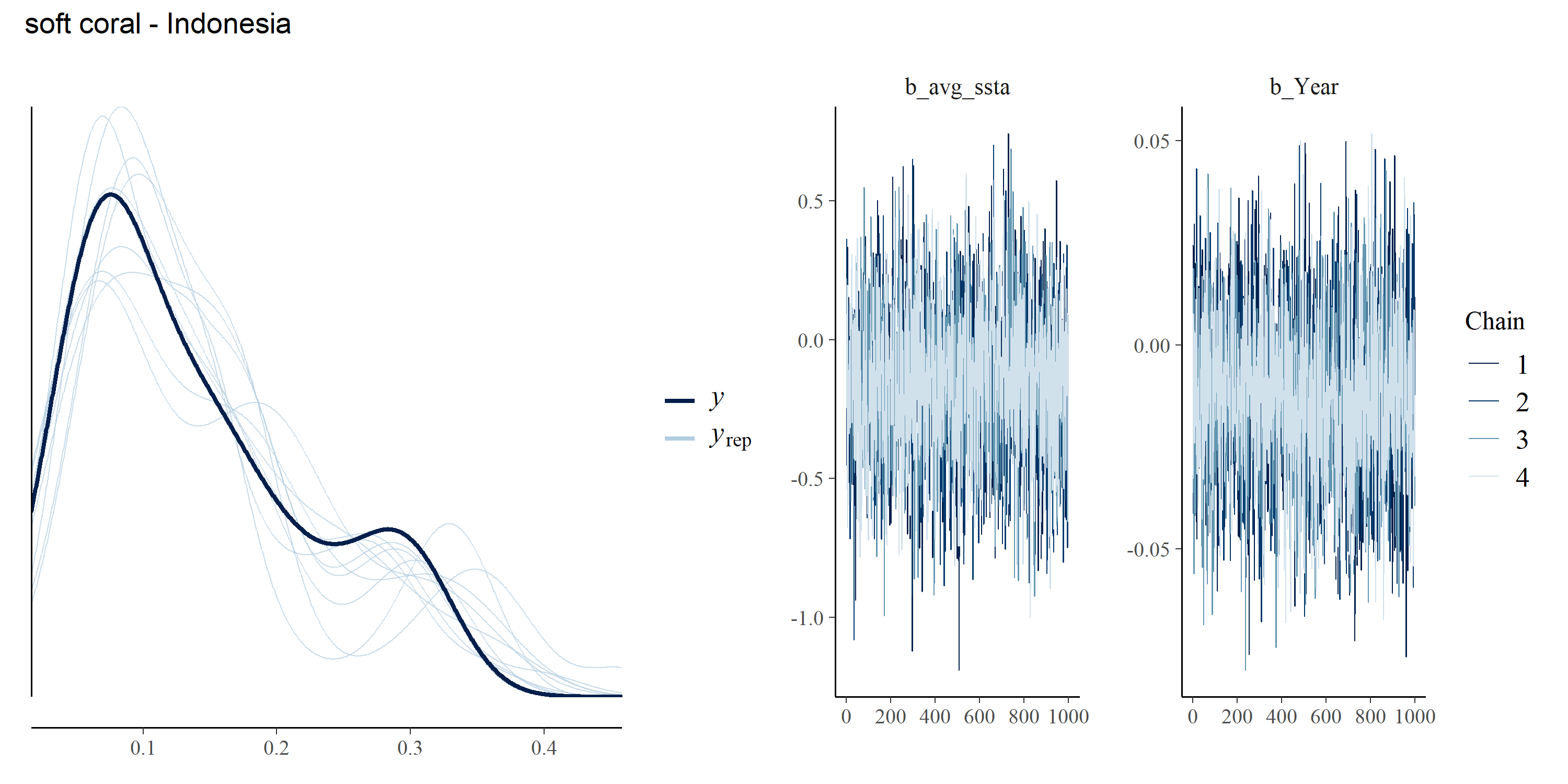


Fig S11. Posterior predictive check and trace plot for soft coral coverage, Indonesia


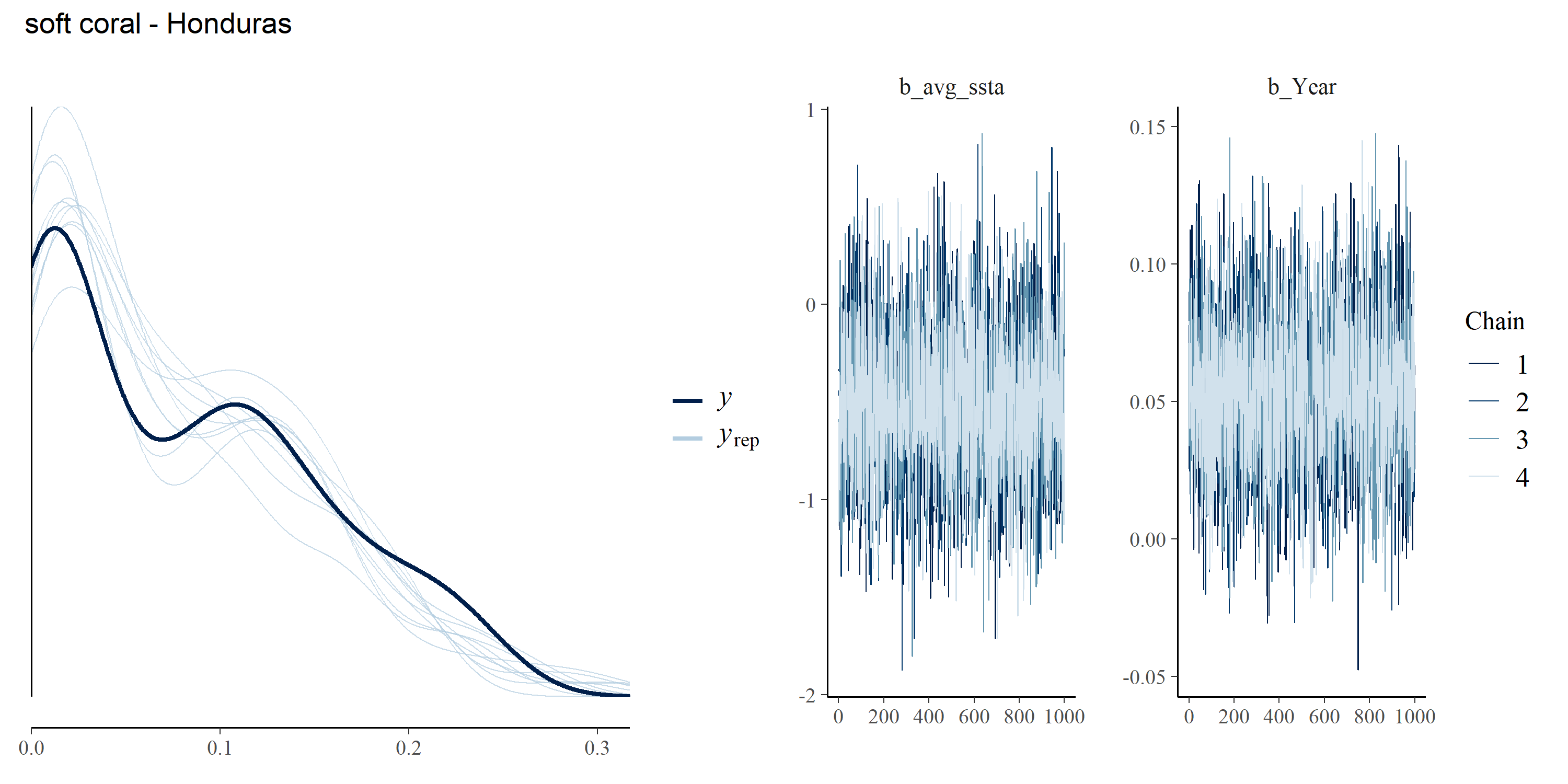


Fig S12. Posterior predictive check and trace plot for soft coral coverage, Honduras


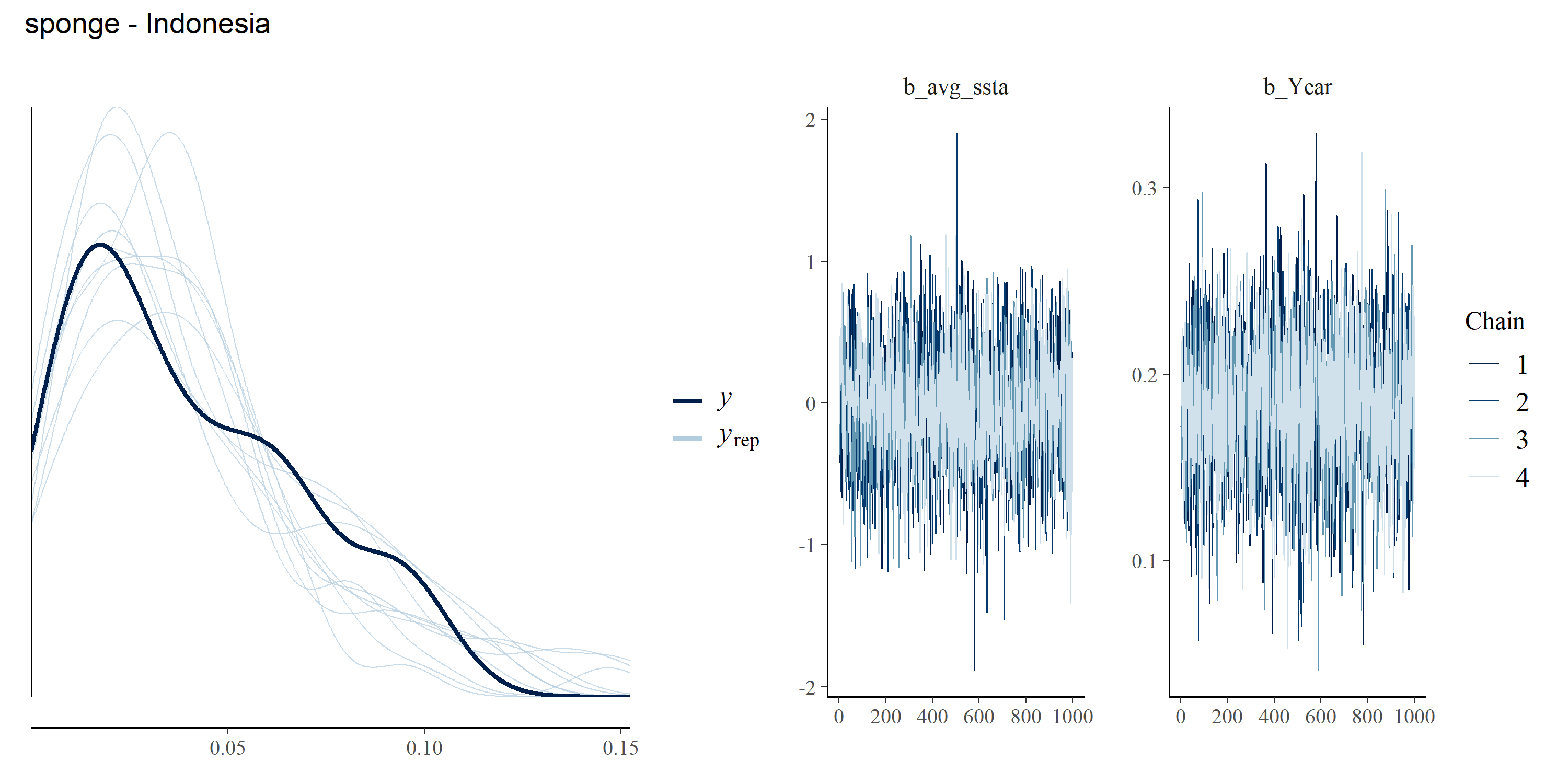


Fig S13. Posterior predictive check and trace plot for Sponge coverage, Indonesia


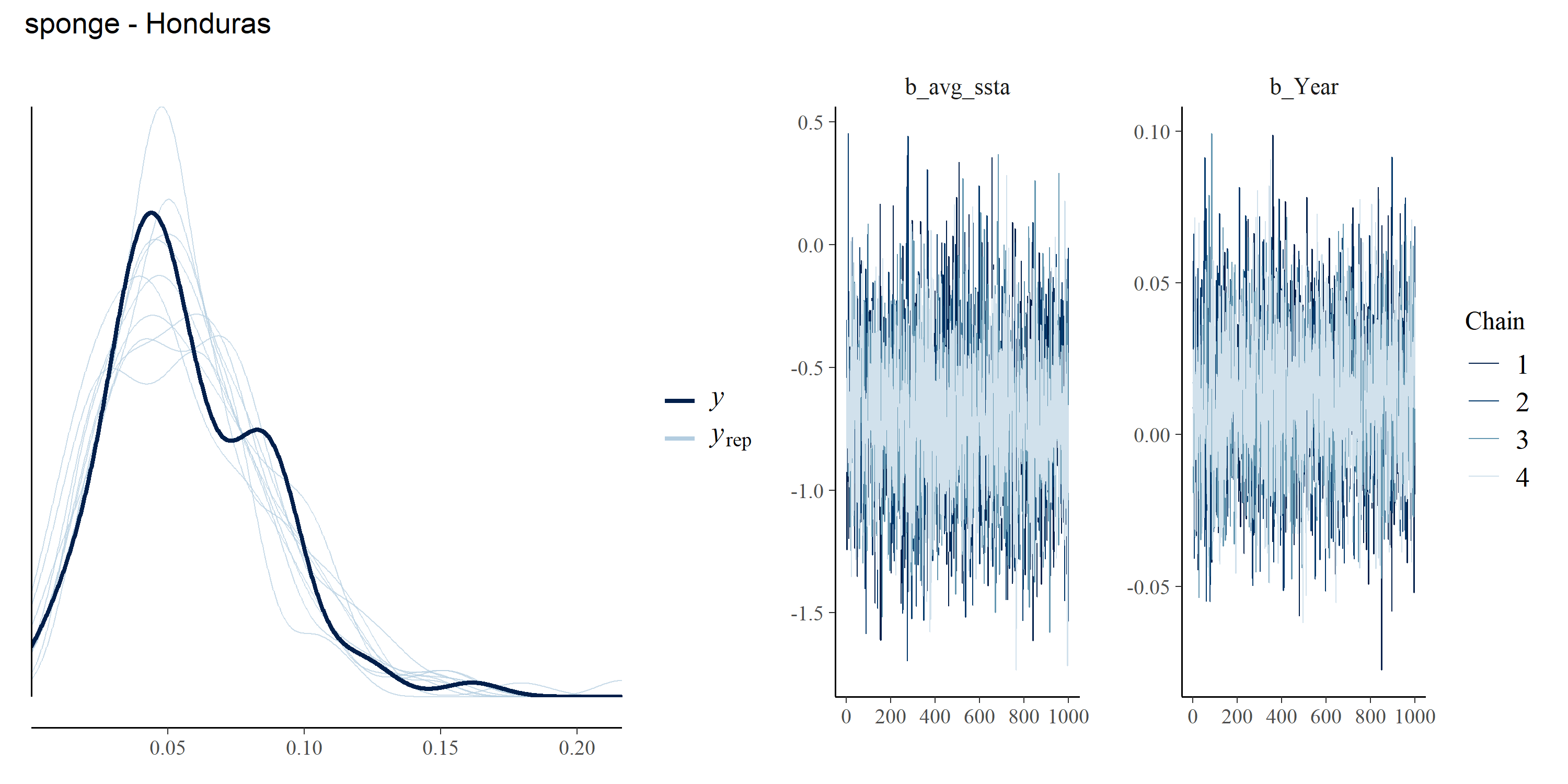


Fig S14. Posterior predictive check and trace plot for Sponge coverage, Honduras
